# Hyperactivation of MEK1 in cortical glutamatergic neurons results in projection axon deficits and aberrant motor learning

**DOI:** 10.1242/dmm.050570

**Published:** 2024-07-02

**Authors:** George R. Bjorklund, Katherina P. Rees, Kavya Balasubramanian, Lauren T. Hewitt, Kenji Nishimura, Jason M. Newbern

**Affiliations:** ^1^ School of Life Sciences, Arizona State University, Tempe, AZ 85287, USA; ^2^ School of Biological and Health Systems Engineering, Arizona State University, Tempe, AZ 85287, USA

**Keywords:** Connectivity, Cortex, Development, RASopathy, Kinase, Axon

## Abstract

Abnormal extracellular signal-regulated kinase 1/2 (ERK1/2, encoded by *Mapk3* and *Mapk1*, respectively) signaling is linked to multiple neurodevelopmental diseases, especially the RASopathies, which typically exhibit ERK1/2 hyperactivation in neurons and non-neuronal cells. To better understand how excitatory neuron-autonomous ERK1/2 activity regulates forebrain development, we conditionally expressed a hyperactive MEK1 (MAP2K1) mutant, MEK1^S217/221E^, in cortical excitatory neurons of mice. MEK1^S217/221E^ expression led to persistent hyperactivation of ERK1/2 in cortical axons, but not in soma/nuclei. We noted reduced axonal arborization in multiple target domains in mutant mice and reduced the levels of the activity-dependent protein ARC. These changes did not lead to deficits in voluntary locomotion or accelerating rotarod performance. However, skilled motor learning in a single-pellet retrieval task was significantly diminished in these *MEK1^S217/221E^* mutants. Restriction of *MEK1^S217/221E^* expression to layer V cortical neurons recapitulated axonal outgrowth deficits but did not affect motor learning. These results suggest that cortical excitatory neuron-autonomous hyperactivation of MEK1 is sufficient to drive deficits in axon outgrowth, which coincide with reduced ARC expression, and deficits in skilled motor learning. Our data indicate that neuron-autonomous decreases in long-range axonal outgrowth may be a key aspect of neuropathogenesis in RASopathies.

## Teaser

**Summary:** MEK1-induced ERK1/2 hyperactivation in developing cortical excitatory neurons is sufficient to decrease long-range axonal outgrowth in mice, which coincides with reduced ARC levels and deficits in skilled motor learning by adulthood.

**Research Simplified**

RASopathies are a group of rare diseases that are caused by the disruption of a fundamental biological pathway in our cells, known as the RAS pathway. This pathway functions as a chain reaction of several different proteins, including MEK1. As RASopathies can disrupt the nervous system, causing different movement- and learning-associated symptoms, it is important to understand how hyperactivation of MEK1 – which would subsequently cause aberrant activation of downstream proteins in the RAS pathway – affects neuronal cells.

In this study, the authors introduced a mutated and hyperactive version of MEK1 into a certain type of neuron in the mouse forebrain. They found that hyperactivation of MEK1 in these neurons impaired skilled motor learning in the mice, which mimics one of the neurological changes of RASopathies. The authors then assessed the neurons in the mice with hyperactive MEK1 and found an inhibition of growth and branching of axons, which are long fibres projected from neurons that allow communication with other cells. This study therefore indicates that disrupted axon growth and branching could be a key driver of some of the neurological changes in individuals with RASopathies. By understanding how these differences arise, future research can devise strategies to study and perhaps treat RASopathies.

## INTRODUCTION

Many extracellular cues important for forebrain development, such as fibroblast growth factors and neurotrophins, activate receptor tyrosine kinases (RTKs) to modulate a wide range of cellular behaviors. Ligand-bound RTKs initiate intracellular signaling via numerous kinase cascades, including the PI3K, PLC, PKC and extracellular signal-regulated kinase 1/2 (ERK1/2) pathways (Lemmon and Schlessinger, 2010). Activation of ERK1/2 (encoded by *Mapk3* and *Mapk1* in mice, respectively) downstream of RTK signaling has been well studied and requires RAS-dependent activation of RAF isoforms and subsequent phosphorylation of MEK1 and MEK2 (encoded by *Map2k1* and *Map2k2*) at two conserved residues in the kinase domain, Ser218/222 and Ser222/226, respectively (Klomp et al., 2021; Lavoie et al., 2020). Activated MEK1/2 then phosphorylates ERK1 and ERK2, which ultimately act upon hundreds of cytosolic and nuclear substrates (Ünal et al., 2017). ERK1/2 signaling has frequently been linked to the control of cell proliferation, differentiation, survival, synaptic plasticity and learning (Atkins et al., 2001; Gómez-Palacio-Schjetnan and Escobar, 2008; Jeanneteau et al., 2010; Kalil and Dent, 2014; Mebratu and Tesfaigzi, 2009; Nowaczyk et al., 2014; Polleux and Snider, 2010; Thomas and Huganir, 2004). However, this broad repertoire obscures the intricate, context-dependent functions of ERK1/2 signaling that are highly contingent on the precise developmental stage, cell type and/or extracellular cue (Santini et al., 2019; Shaul and Seger, 2007). Developing neurons in particular exhibit complex, subtype-specific responses to modifications in ERK1/2 activity that are not fully understood (Ryu and Lee, 2016).

Key regulators in the RTK-ERK1/2 signaling network are frequently mutated in a family of developmental syndromes known collectively as the ‘RASopathies’ (Jindal et al., 2015; Kim and Baek, 2019; Rauen, 2013, 2022). Well-defined variants have been identified in individual syndromes within the RASopathy superfamily, such as Noonan syndrome (NS), neurofibromatosis type 1 (NF1), Costello syndrome and cardio-facio-cutaneous syndrome. NS and NF1 are common RASopathies with a combined frequency of ∼1 in 2000 births (Rauen, 2013; Tidyman and Rauen, 2009). However, >50 distinct RASopathy variants in over ten different genes within the RTK-ERK1/2 network have been reported (Brown et al., 2012; Gutmann et al., 2012; Jindal et al., 2015; Kaul et al., 2015; Rauen, 2022). Upstream RASopathy variants in the NF1, SHP2 (encoded by *Ptpn11*) and RAS proteins have been shown to modulate PI3K/Akt, PKC and/or Rho/ROCK activity, in addition to the ERK1/2 pathway (Anastasaki and Gutmann, 2014; Brown et al., 2012; Castellano and Downward, 2011; Kaul et al., 2015). ERK1/2 hyperactivation is, however, thought to be a core, common feature of nearly all RASopathies. Thus, pharmacological inhibitors of ERK1/2 regulators have been an important focus for therapeutic development (Guilding et al., 2007; López-Juárez et al., 2017; Nakamura et al., 2009; Wang et al., 2012; Wu et al., 2011). ERK1/2 also represents a convergent target downstream of multiple genetic risk factors for autism and is abnormally activated in models of fragile X and 16p11.2 deletion syndromes (Chung et al., 2021; Osterweil et al., 2013; Wen et al., 2016). Therefore, understanding the cell-specific effects of ERK1/2 activity in the developing forebrain may shed light on potential neuropathological processes in multiple developmental disorders (Bernardino et al., 2022; Li et al., 2005; Roskoski, 2019).

Magnetic resonance imaging (MRI)-based studies suggest that differences in both cortical structural and functional connectivity are a critical aspect of intellectual disability and cognitive changes in RASopathies and autism (Aydin et al., 2016; Chang et al., 2016; Fattah et al., 2021; Filippi et al., 2013; Ibrahim et al., 2017; Owen et al., 2018; Tomson et al., 2015; Zamboni et al., 2007). Changes in motor cortex function have been detected in RASopathies, and individuals with NS note that treatments for cognitive and musculoskeletal abnormalities are a high priority (Bernardino et al., 2022; Doherty et al., 2023; Tiemens et al., 2023). Yet, it is difficult to discern whether functional changes are a result of aberrant signaling in neurons or in myelinating oligodendrocytes, or complex interactions during development. Studies have shown a specific role for ERK1/2 signaling in regulating rodent myelinating glial development *in vivo* (Ishii et al., 2012, 2013; Newbern et al., 2011), and pharmacological MEK1/2 inhibition rescues myelination deficits caused by RASopathic signaling in mature oligodendrocytes (López-Juárez et al., 2017; Mayes et al., 2013). The effects of upstream RASopathy variants in the SynGAP1, NF1 and RAS proteins that activate multiple downstream cascades, selectively in neurons, have been explored (Clement et al., 2012; Cui et al., 2008; Kim et al., 2023; Rumbaugh et al., 2006). However, the neuron-autonomous effects of downstream ERK1/2 hyperactivation in developing cortical excitatory circuits are less clear. We previously reported subtype-specific functions for ERK1/2 in the development of cortical inhibitory and excitatory neurons with a series of Cre-dependent conditional mouse lines. Deletion of *Mek1* and *Mek2* in postmitotic excitatory neurons resulted in reduced corticospinal axon extension into the lumbar spinal cord, followed by death of a subset of layer V CTIP2 (encoded by *Bcl11b*)-positive neurons in the neonatal motor cortex (Xing et al., 2016). In contrast, hyperactivation of ERK1/2 signaling in excitatory neurons via overexpression of a Cre-dependent, phosphomimic MEK1^S217/221E^ construct did not alter CTIP2^+^ density but led to reductions in corticospinal elongation by postnatal day (P) 3 (Xing et al., 2016).

Given the marked excitatory neuron subtype-specific outcomes associated with modifying ERK1/2 activity, we sought to understand whether MEK1 hyperactivation in excitatory neurons during development is sufficient to alter cortical axon outgrowth to multiple targets and ultimately affect cognition. We used a *Nex:Cre* mouse to conditionally express a hyperactive *MEK1*^*S217/221E*^ construct in newborn, postmitotic cortical excitatory neurons, but not in oligodendrocytes or cortical inhibitory neurons (Goebbels et al., 2006; Krenz et al., 2008). *MEK1*^*S217/221E*^ selectively drives downstream hyperactivation of ERK1/2, independent of directly manipulating parallel RTK-linked cascades. Moreover, *Nex:Cre*-directed expression minimizes cellular contributions from glia and inhibitory neurons known to be modified by changes in ERK1/2 signaling (Angara et al., 2020; Holter et al., 2021; Kang and Lee, 2019; Knowles et al., 2023a,b). This approach will help identify the excitatory neuron-autonomous functions of selective hyperactivation of MEK-ERK1/2 signaling during long-range cortical circuit formation.

Our results show that conditional *MEK1*^*S217/221E*^ overexpression leads to a persistent increase in phosphorylated ERK1/2 (pERK1/2) levels in axonal projections, which coincides with reduced motor cortex-derived axon arborization in intracortical and subcortical target domains. Moreover, mutant cortices exhibited reduced expression of a canonical immediate early protein, ARC, and deficits in learning a single-pellet retrieval task (Guo et al., 2015; Kawai et al., 2015; Peters et al., 2017; Whishaw et al., 1993). To test whether these deficits were cortical layer V autonomous, we crossed the *MEK1*^*S217/221E*^ mouse line with a *Rbp4:Cre* mouse. Viral tracing analysis of contralateral corticocortical, corticostriatal and corticospinal projections in *Rbp4:Cre*; *MEK1*^*S217/221E*^ mutants revealed a significant decrease in axonal innervation, but no deficit in skilled motor learning. Collectively, these results indicate that excitatory neuron-specific hyperactivation of MEK1 in the cortex is sufficient to disrupt the development of cortical axonal projections, the expression of ARC and select aspects of skilled motor learning.

## RESULTS

### Hyperactivation of MEK1 increases levels of pERK1/2 in cortical excitatory neuron axons

**Fig. 1.**
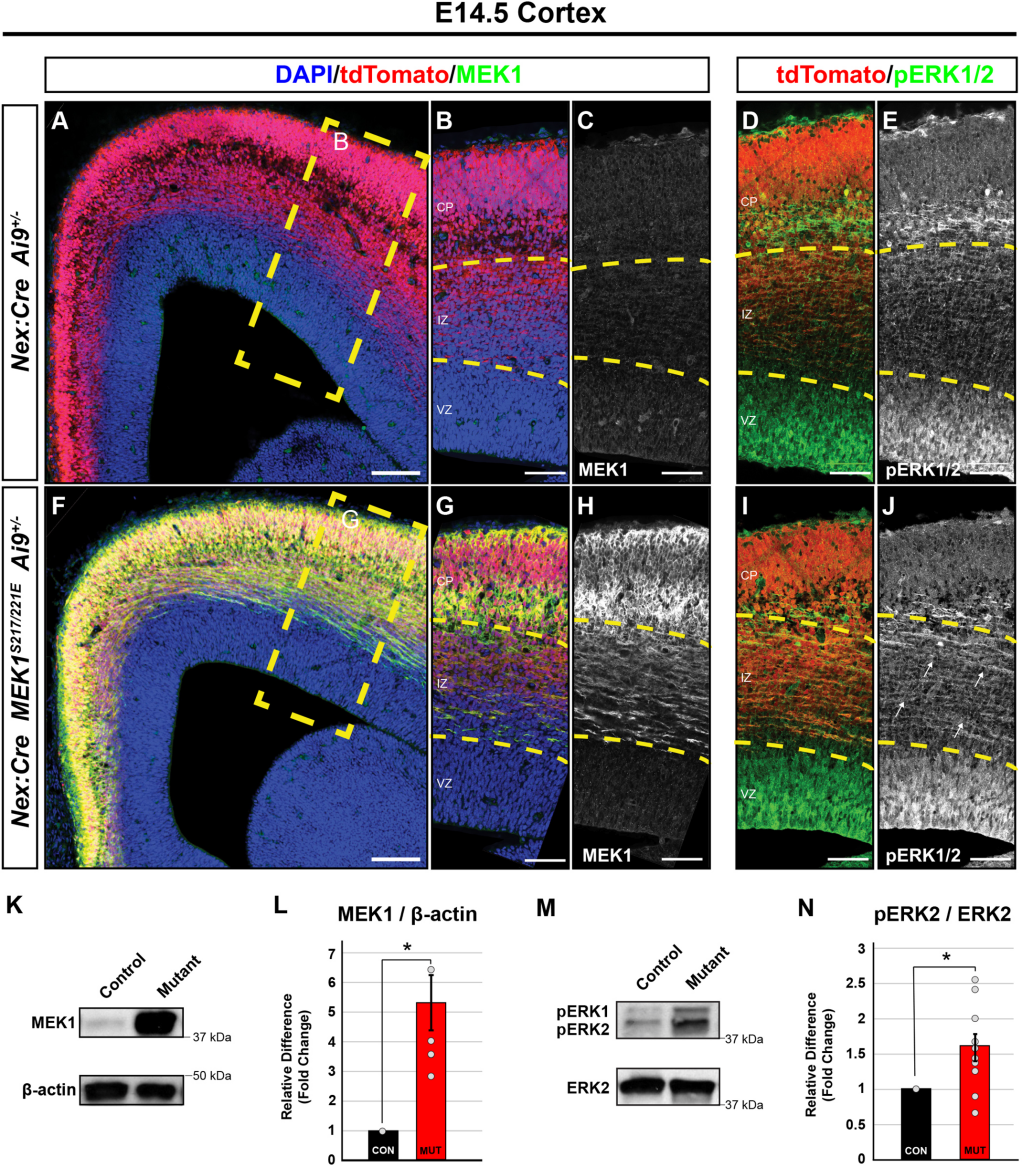
**Selective MEK1 hyperactivation in immature glutamatergic neurons results in increased levels of pERK1/2 by E14.5.** (A-J) Immunolabeling of embryonic day (E) 14.5 *Nex:Cre; MEK1^S217/221E^; Ai9^+/−^* mutant cortices showed a substantial increase of MEK1 expression in the intermediate zone (IZ) and cortical plate (CP) of mutants (F-H) compared to that in *Nex:Cre; Ai9^+/−^* controls (A-C) (*n*=4). tdTomato^+^ axons within the IZ of mutants (I,J, white arrows) exhibited a qualitative enrichment in levels of phosphorylated ERK1/2 (pERK1/2) relative to that in controls (D,E) (*n*=4). We did not detect a notable difference in the levels of pERK1/2 in the ventricular zone (VZ), where recombination is essentially absent in these mice. The integrated density (mean gray value×mm^2^) of the CP+IZ in panels C,H,E,J is 1.36, 4.70, 4.46 and 4.10, respectively. Scale bars: 100 µm (A,F); 60 µm (B-E,G-J). (K-N) Western blots of E14.5 dorsal cortical lysates (K,M) revealed a highly significant increase in MEK1 (L) (*n*=5, two-tailed unpaired Student's *t*-test, *P=0.002) and pERK2 (*n*=10, two-tailed unpaired Student's *t*-test, *P=0.005) levels in mutants compared to those in controls. Data are shown as mean±s.e.m.

To investigate the developmental effects of excitatory neuron-autonomous hyperactive MEK-ERK1/2 signaling within the cortex *in vivo*, we generated mice expressing *Nex:Cre*, Cre-dependent hyperactive *MEK1*^*S217/221E*^ and Cre-dependent tdTomato/RFP (*Ai9*^+/−^) (Goebbels et al., 2006; Krenz et al., 2008; Madisen et al., 2010). In the cortex of these mice, Cre recombinase is selectively expressed shortly after excitatory neuron specification during mid-embryogenesis in pyramidal neurons, which then populates the cortical laminal layers II-VI (Goebbels et al., 2006; Xing et al., 2016). We have previously shown Cre-mediated recombination in excitatory neuron lineages expressing the master transcription factors SATB2 and CTIP2 in *Nex:Cre* mice, but not in neural stem cells, non-neuronal cells and inhibitory GABAergic interneuron lineages (Xing et al., 2016). As expected, embryonic day (E) 14.5 *Nex:Cre; Ai9^+/−^* forebrains showed Cre-dependent tdTomato expression in excitatory neuron somas in the cortical plate (CP) and excitatory neuron axons in the intermediate zone (IZ) (Fig. 1A,B). Importantly, substantial Cre-dependent MEK1 overexpression was also apparent in *Nex:Cre; MEK1*^*S217/221E*^*; Ai9^+/−^* mutant cortices in presumptive cortical excitatory neuron somas in the CP and axon projections within the IZ (Fig. 1F-H) compared to controls (Fig. 1A-C). Semi-quantitative assessment of MEK1 expression in western blots of dorsal cortical lysates from E14.5 *Nex:Cre; MEK1*^*S217/221E*^ embryos demonstrated a 5.29±0.93-fold increase relative to its expression in controls (mean±s.e.m., *n*=5, two-tailed unpaired Student's *t*-test, *P=0.002) (Fig. 1K,L).

MEK1^S217/221E^ hyperphosphorylates ERK1/2 in cell-free assays and in culture, but the extent of downstream effects *in vivo* has been shown to vary in different tissue types (Alessi et al., 1994; Bueno, 2000; Cowley, 1994; Klesse et al., 1999; Krenz et al., 2008; Lajiness et al., 2014; Li et al., 2012). We directly assessed whether *MEK1*^*S217/221E*^ overexpression targeted toward developing cortical excitatory neurons drives early and persistent increases in pERK1/2 levels *in vivo*. The ventricular zone of forebrain coronal sections, a region lacking *Nex:Cre*-mediated recombination, exhibited comparable levels of pERK1/2 immunolabeling in control and mutant embryos (Fig. 1D,E,I,J). However, immunolabeling for pERK1/2 in *Nex:Cre; MEK1*^*S217/221E*^*; Ai9^+/−^* embryos revealed a relative qualitative enrichment of pERK1/2 in nascent tdTomato^+^ projections within the IZ, presumably cortical excitatory neuron axons, relative to that in controls (Fig. 1I,J, white arrows). Western blotting analyses of E14.5 dorsal cortical lysates from *Nex:Cre;MEK1*^*S217/221E*^ mutant mice showed a significant 1.61±0.19-fold increase in pERK2 levels compared to those in controls (mean± s.e.m., *n*=10, two-tailed unpaired Student's *t*-test, *P=0.005) (Fig. 1M,N).

We asked whether the increased MEK1 and pERK1/2 levels in mutants persisted into adulthood. Adult *Nex:Cre; MEK1^S217/221E^* cortical lysates revealed a statistically significant 1.61±0.2-fold increase in MEK1 expression over that in controls (mean±s.e.m., *n*=3, two-tailed unpaired Student's *t*-test, *P=0.04) (Fig. S1A,B). The proportion of excitatory neurons in whole-adult cortical lysates is relatively less than that at E14.5 (Goebbels et al., 2006) and may contribute to the reduced fold increase in MEK1 in adult mutants compared to that in E14.5 mutants. Interestingly, we did not detect a significant difference in pERK1/2 levels between *Nex:Cre; MEK1^S217/221E^* and control whole cortical lysates (mean±s.e.m., *n*=3, two-tailed unpaired Student's *t*-test, *P=0.83) (Fig. S1C,D).

**Fig. 2.**
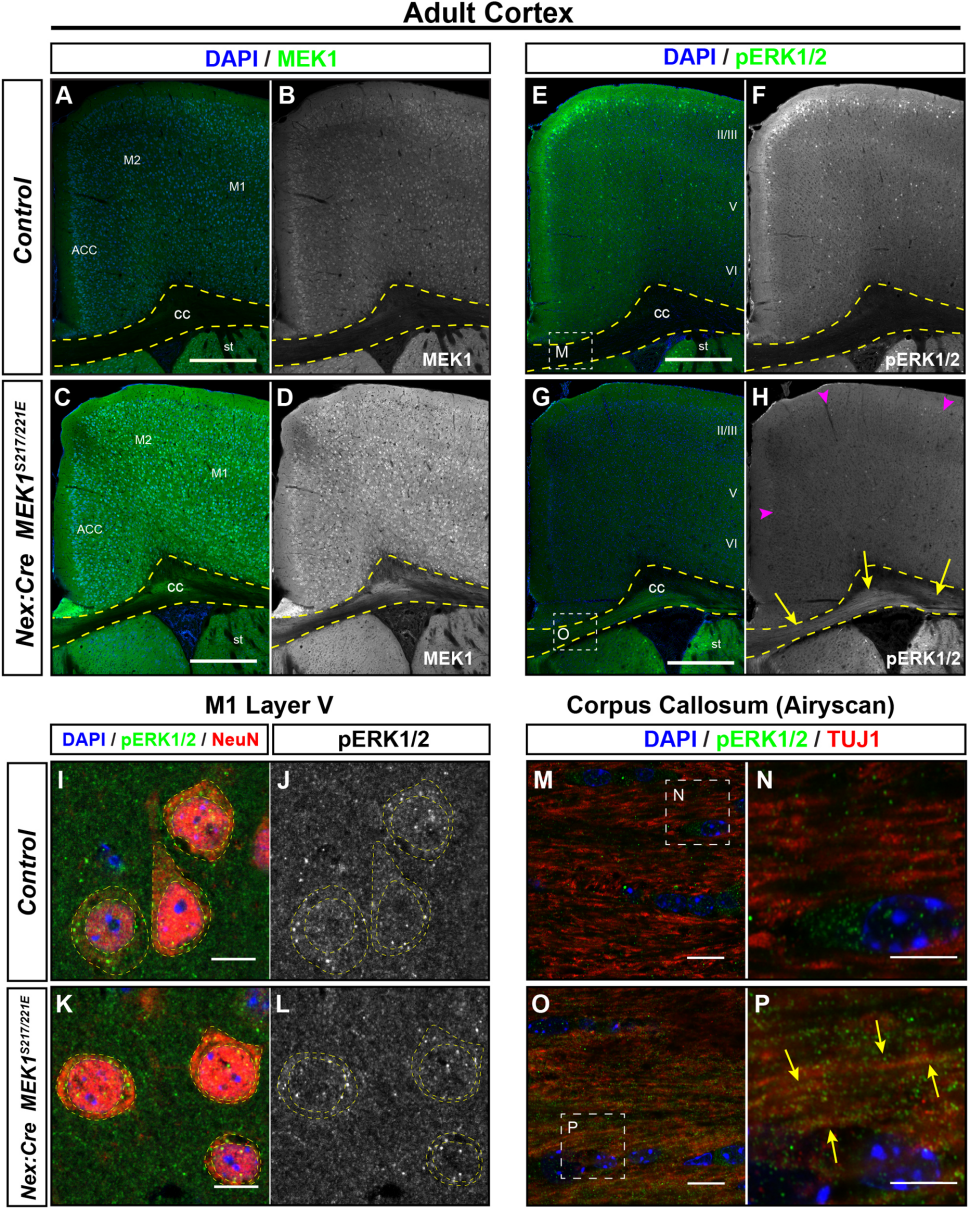
**Adult *Nex:Cre; MEK1^S217/221E^* cortical excitatory neurons exhibit compartment-specific changes in pERK1/2 levels.** (A-H) An increase in MEK1 immunolabeling was apparent in the cortical grey matter and corpus collosum of adult *Nex:Cre; MEK1^S217/221E^* mice (C,D) relative to that in controls (A,B). Within the mutant grey matter, we noted a significant decrease in pERK1/2-labeled neurons in layers II/III (G,H, magenta arrowheads) compared to those in controls (E-F) (*n*=6, quantitation in Fig. S1A). The integrated density (mean gray value×mm^2^) of pERK1/2 labeling in cortical grey matter is 60.1 and 48.4 in panels F and H, respectively. In contrast, pERK1/2 immunolabeling was qualitatively increased in the corpus callosum (CC, dashed yellow outlines) of adult mutants (G,H, yellow arrows, the CC integrated density in H is 7.3) compared to that in controls (E,F, the CC integrated density in F is 2.5). ACC, anterior cingulate cortex; M1, primary motor cortex; M2, secondary motor cortex; st, striatum. (I-L) Representative high-resolution confocal images of NeuN/DAPI-labeled neurons in M1 layer V (I-L, dashed yellow outlines) revealed a decrease in cytoplasmic and nuclear levels of pERK1/2 in mutants (K,L) compared to those in controls (I,J) (*n*=4, individual neuron quantitation in Fig. S1B). (M-P) Confocal Airyscan imaging was used to better resolve individual TUJ1^+^ projections in the corpus callosum, likely derived from callosally projecting excitatory neurons. We detected a modest increase in pERK1/2 colocalization with TUJ1 in mutants (O,P, yellow arrows) compared to that in controls (M,N) (*n*=4, quantification in Fig. S2C). Scale bars: 500 µm (A-H); 10 µm (I-L); 2 µm (M,O); 1 µm (N,P).

We next examined the levels of MEK1 and pERK1/2 in adult cortical neuron somas within the grey matter and in white matter regions enriched in excitatory projection neuron axons. Relative to that in controls (Fig. 2A,B), MEK1 immunolabeling was substantially increased in mutant presumptive excitatory neurons somas, in addition to the corpus callosum and other subcortical tracts (Fig. 2C,D). Control forebrains immunolabeled for pERK1/2 showed the expected enrichment in a subset of neuronal pyramidal-appearing somas in layers II/III, with relatively lower levels in the corpus callosum (Fig. 2E,F) (Hart and Balleine et al., 2016; Lee et al., 2021). Interestingly, in *Nex:Cre; MEK1^S217/221E^* mutant mice, we observed a significant decrease in the density of pERK1/2^+^ layer II/III neurons across much of the dorsal cortex compared to that in controls (Fig. 2G,H, magenta arrowheads). Quantification of the number of pERK1/2^+^ layer II/III neurons in the primary motor cortex (M1) per section also revealed a decrease in the mutants (*n*=6, two-tailed unpaired Student's *t*-test, *P*=0.002) (Fig. S2A).

The NeuN antigen, recognizing the protein RBFOX3, labels both cortical GABAergic interneurons and glutamatergic neurons. However, ∼70-80% of cortical neurons are glutamatergic, which can be distinguished by their large somal size relative to that of GABAergic neurons (Serradas et al., 2022), as well as their relatively higher level of NeuN labeling (Chattopadhyaya et al., 2004). We performed semi-quantitative high-resolution analyses of pERK1/2 immunolabeling intensity in confocal images of layer V neurons on large, intensely labeled NeuN^+^ profiles with a pyramidal morphology to enrich our analysis for excitatory neurons. Our results revealed a reduction in pERK1/2 immunolabeling intensity in the cytoplasm and the nuclei of mutant layer V neurons compared to that in controls (*n*=68 control and 83 mutant neurons from four mice per genotype, two-tailed unpaired Student's *t*-test, *P*<0.01) (Fig. 2I-L; Fig. S2B). These data further suggest that the levels of somal/nuclear pERK1/2 are unexpectedly reduced in adult *Nex:Cre; MEK1^S217/221E^* excitatory neurons.

In contrast to the somal compartment, we noted a qualitative increase in pERK1/2 immunolabeling in a number of mutant forebrain white matter tracts, such as the corpus callosum (Fig. 2H, yellow arrows), fimbria and stria terminalis (Fig. S2D-G), which are enriched in axons derived from *Nex:Cre*-expressing excitatory populations in the cortex, hippocampus and amygdala, respectively. High-resolution analysis of pERK1/2 colocalization with TUJ1, a marker of axons, in the corpus callosum demonstrated the presence of pERK1/2 in axons and a modest increase in colocalization in mutant axons compared to that in controls (*n*=4, two-tailed unpaired Student's *t*-test, *P*=0.054) (Fig. 2M-P yellow arrows; Fig. S2C). Collectively, these data suggest that excitatory neuron-autonomous *MEK1^S217/221E^* expression results in persistent hyperactivation of ERK1/2 in the axonal compartment of multiple forebrain projection subtypes.

### MEK1^S217/221E^ expression disrupts corticocortical and corticostriatal axon arborization

**Fig. 3.**
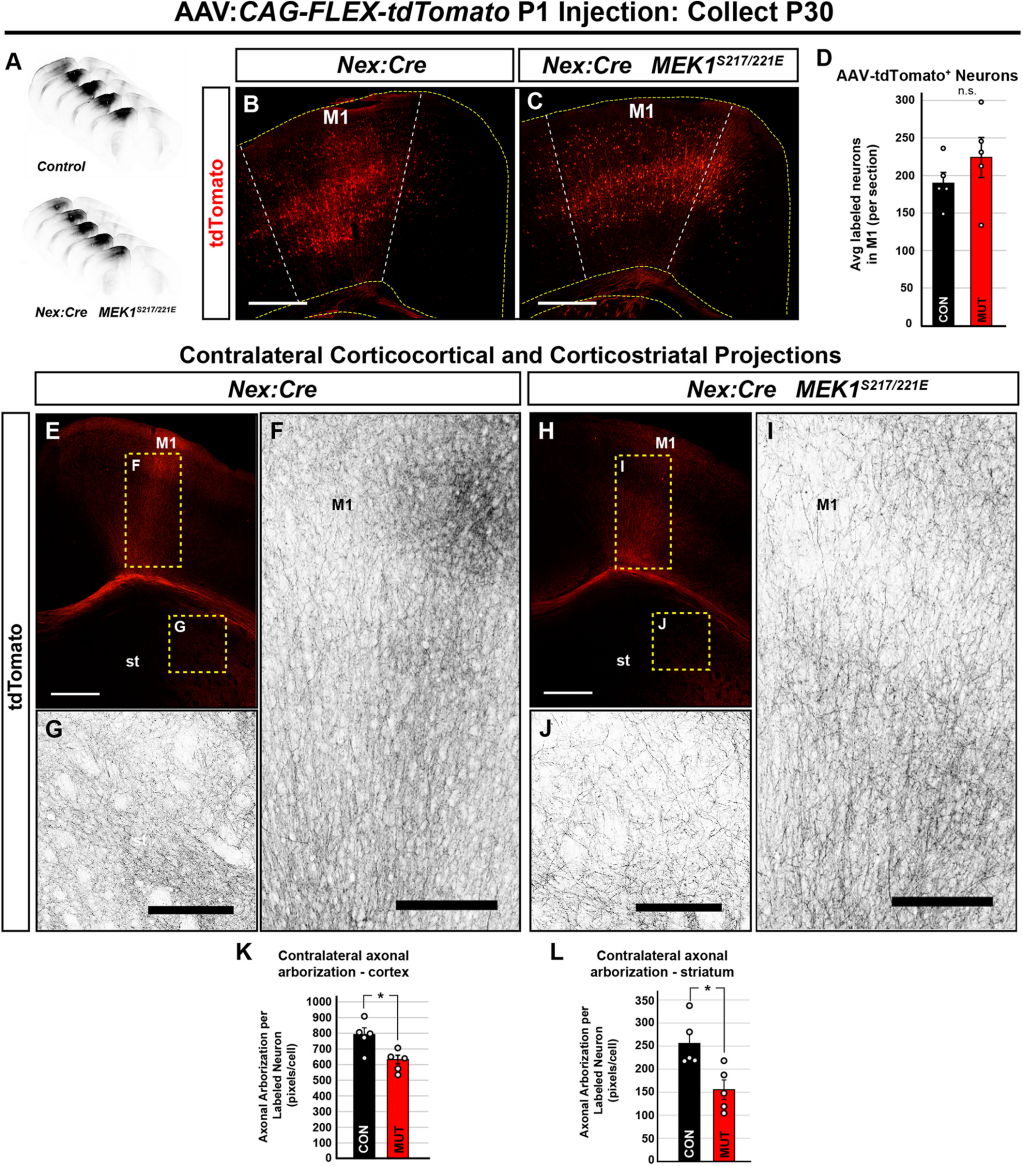
***Nex:Cre; MEK1^S217/221E^* mice exhibit disrupted contralateral corticocortical and corticostriatal arborization.** (A-D) The adeno-associated viral vector AAV:*CAG-Flex-tdTomato* was injected into the left primary motor cortex (M1) at postnatal day (P) 1 and brains were collected at P30, as shown in the rostral to caudal serial sections surrounding the injection site (A) and representative images of the injected region in the M1 of the control (B) and *Nex:Cre; MEK1^S217/221E^* (C) forebrains. Dashed white and yellow lines in B,C show the boundaries of M1 and the cortex, respectively. No significant difference (n.s.) in the average density of tdTomato-labeled cells was detected between *Nex:Cre; MEK1^S217/221E^* and control mice (mean±s.e.m., *n*=5, two-tailed unpaired Student's *t*-test, *P*=0.2839) (D). (E-L) Representative confocal images of contralateral corticocortical and corticostriatal (st, striatum) tdTomato-labeled axonal arborization in *Nex:Cre; MEK1^S217/221E^* (H,J) and control mice (E,G). *Nex:Cre; MEK1^S217/221E^* mice showed a 19.57±4.30% reduction of normalized intracortical axon arborization (I) compared to that in control mice (mean±s.e.m., *n*=5, two-tailed unpaired Student's *t*-test, *P=0.014) (F, quantified in K). Cortical axon arborization in the mutant dorsolateral striatum was reduced by 37.50±9.14% (J) compared to that in control mice (mean±s.e.m., *n*=5, two-tailed unpaired Student's *t*-test, *P=0.013) (G, quantified in L). Scale bars: 500 µm (B,C,E,H); 100 µm (F,G,I,J).

We previously reported a reduction in corticospinal tract (CST) elongation in *Nex:Cre; MEK1^S217/221E^* mice by P3, which persisted to P30 (Xing et al., 2016). Here, we asked whether MEK1^S217/221E^ expression is sufficient to reduce axon arborization in two other target domains, the contralateral cortex and the striatum. To unilaterally label developing neurons and their axons, a Cre-dependent tdTomato/RFP adeno-associated viral vector (AAV:*CAG-FLEX-tdTomato*) was injected into M1 at P1 (Fig. 3A). There was no significant difference in the density of tdTomato-labeled cells per section between *Nex:Cre; MEK1^S217/221E^* (224.73±26.73) and control (189.96±14.20) motor cortices (mean±s.e.m., *n*=5, two-tailed unpaired Student's *t*-test, *P*=0.284) (Fig. 3B-D). To estimate axonal arborization, tdTomato-labeled pixels in contralateral M1 and striatum were quantified. There was a statistically significant reduction of contralateral corticocortical arborization in *Nex:Cre; MEK1^S217/221E^* mice (631.68±27.51 labeled pixels/transduced cell) compared to that in controls (791.79±42.88 labeled pixels/transduced cell) when normalized to number of transduced cells in M1 (mean±s.e.m., *n*=5, two-tailed unpaired Student's *t*-test, *P*=0.014) (Fig. 3E,F,H,I,K). Contralateral corticostriatal projections into the dorsolateral striatum were also significantly reduced in the *Nex:Cre; MEK1^S217/221E^* mice (control, 255.67±23.64 labeled pixels/transduced cell; mutant: 155.44±21.18 labeled pixels/transduced cell; mean±s.e.m., *n*=5, two-tailed unpaired Student's *t*-test, *P*=0.013) (Fig. 3E,G,H,J,L). Taken together with our previous findings, cortical glutamatergic neuron-autonomous *MEK1^S217/221E^* expression appears sufficient to hyperactivate ERK1/2 during axonal development and reduce arborization into multiple long-range targets.

### ARC protein levels are reduced by MEK1 hyperactivation

**Fig. 4.**
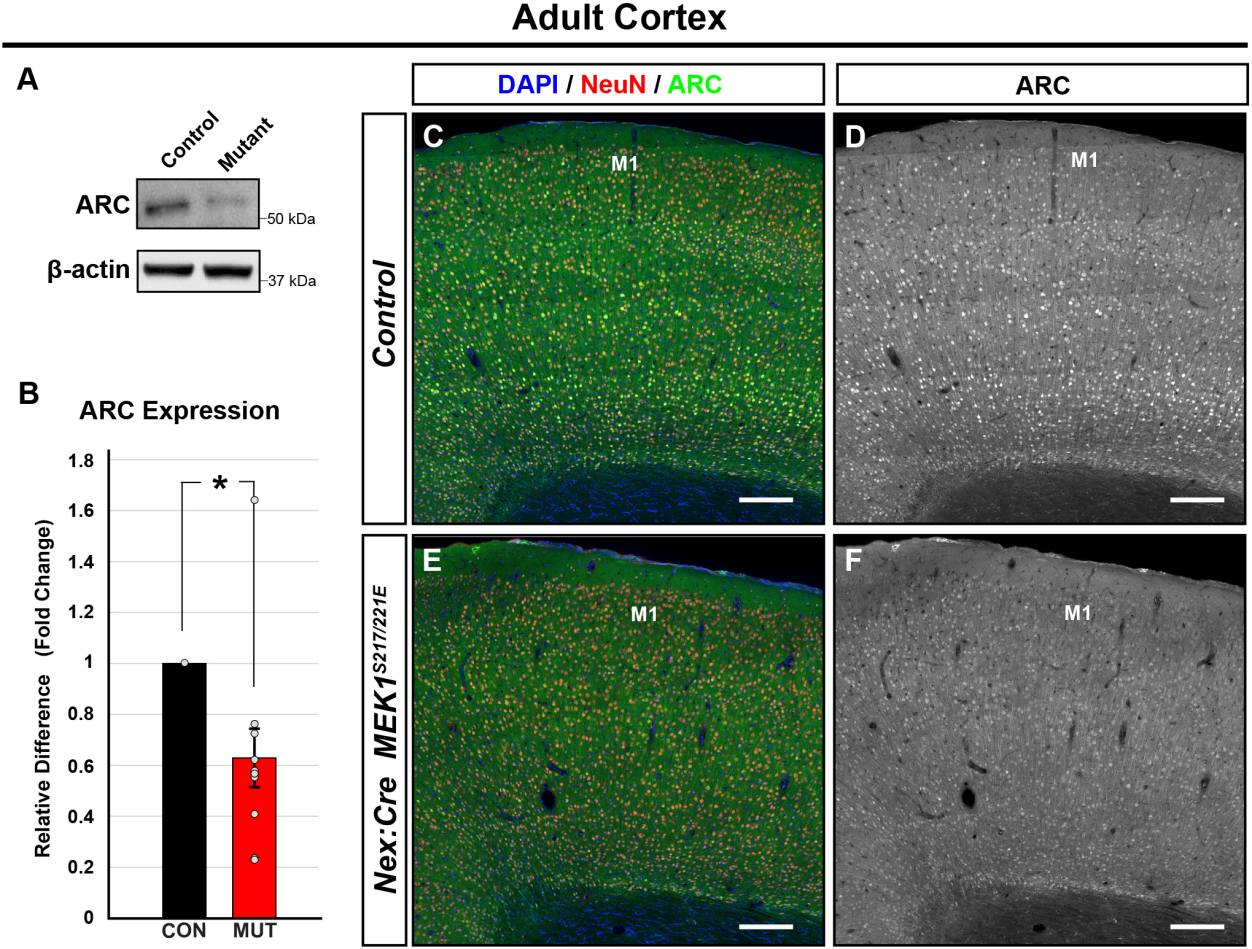
**MEK1^S217/221E^ reduces cortical ARC protein expression.** (A,B) Western blots of adult cortical lysates (A) and quantification of band intensities (B) showed a significant reduction in ARC protein levels in *Nex:Cre; MEK1^S217/221E^* mutants compared to those in control mice (mean±s.e.m., *n*=11, two-tailed unpaired Student's *t*-test, *P<0.005). (C-F) Immunolabeling revealed a qualitative reduction of ARC in *Nex:Cre; MEK1^S217/221E^* cortices (E,F) compared to that in controls (C,D) (*n*=5). The integrated density (mean gray value×mm^2^) of ARC in D and E is 96.6 and 72.3, respectively. M1, primary motor cortex. Scale bars: 200 µm.

ERK1/2 is a known regulator of glutamatergic signaling- and activity-dependent plasticity (Bramham et al., 2010; Thomas and Huganir, 2004). Whether *MEK1^S217/221E^* expression alters circuit-level cortical activity in adults is unclear. Activity-regulated cytoskeleton-associated gene (*Arc*) is an immediate early gene triggered by glutamate receptor signaling via ERK1/2 and other pathways (Cao et al., 2015; Chotiner et al., 2010; Correa et al., 2012; Ebert and Greenberg, 2013; Pintchovski et al., 2009; Ying et al., 2002). Our data show that axonal arborization as well as somal and nuclear levels of pERK1/2 are reduced in *Nex:Cre; MEK1^S217/221E^* mutants, indicating that glutamatergic drive may be diminished. Indeed, western blots of adult whole cortical lysates revealed a significant 37±11% decrease in ARC expression in *Nex:Cre; MEK1^S217/221E^* mice compared to that in controls (*n*=11, two-tailed unpaired Student's *t*-test, *P*<0.005) (Fig. 4A,B). Immunolabeling further indicated a widespread decrease in cortical ARC levels *in vivo* (*n*=4) (Fig. 4C-F). These findings support that developmental expression of hyperactive MEK1 in glutamatergic neurons reduces cortical ARC expression *in vivo.*

### Motor skill acquisition is disrupted in *Nex:Cre; MEK1^S217/221E^* mice

**Fig. 5.**
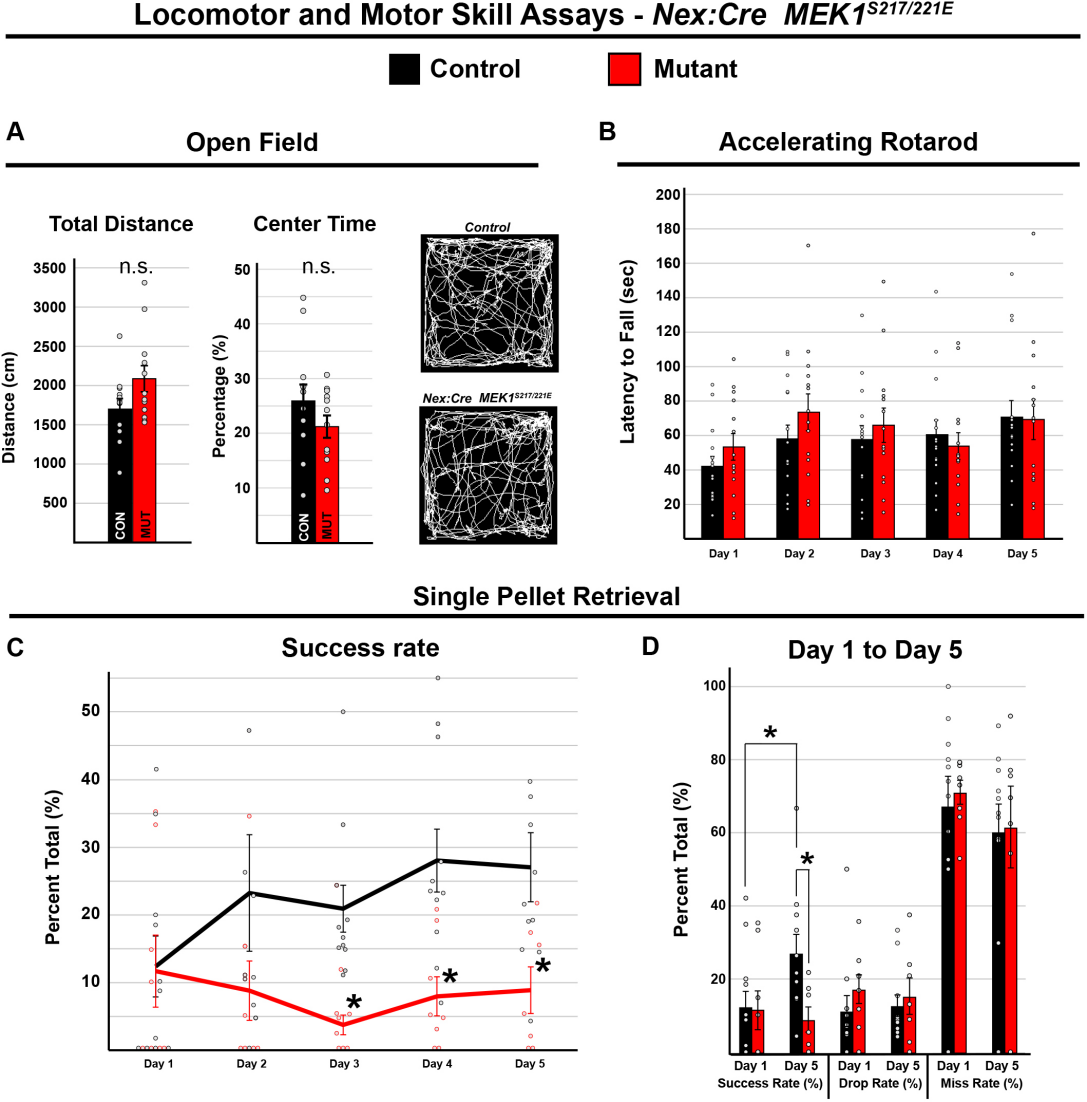
**Motor skill acquisition is disrupted by MEK1 hyperactivation in cortical glutamatergic neurons.** (A) Open-field testing of *Nex:Cre; MEK1^S217/221E^* (*n*=12) and control (*n*=12) mice revealed no significant difference (n.s.) in distance traveled (mean±s.e.m., two-tailed unpaired Student's *t*-test, *P*=0.075) or percentage of time spent in the center of the arena (mean±s.e.m., two-tailed unpaired Student's *t*-test, *P*=0.21). (B) Accelerating rotarod testing revealed a significant effect of day {two-way repeated measures ANOVA, [*F*_(4, 27)_=6.144, *P*<0.001]} over 5 days of testing, but no significant effect of genotype {mean±s.e.m., *n*=15 controls and 14 mutants, [*F*_(1, 27)_=0.246, *P*=0.624]}. (C,D) In the single-pellet retrieval task, the control success rate significantly improved over 5 days from 12.39±4.50 to 27.06±5.11% [mean±s.e.m., least significant difference (LSD) post hoc two-tailed unpaired *t*-test for day 1 versus day 5, *P=0.016, *n*=11]. The *Nex:Cre; MEK1^S217/221E^* mutant mice did not show significant improvement and exhibited a significantly lower success rate relative to that for controls during the final 3 days of testing {mean±s.e.m., *n*=11 controls and 8 mutants, two-way repeated measures ANOVA [*F*_(1,16)_=11.04, *P*=0.004], LSD post hoc test, *P<0.05}.

Common phenotypes in individuals with RASopathies include hypotonia, changes in motor coordination and altered motor learning (Blüthgen et al., 2017; Davis et al., 2000; Monje et al., 2005). We thus asked whether hyperactivation of MEK1 in *Nex:Cre*-expressing cells was sufficient to drive changes in adult mouse motor behavior in open-field, accelerating rotarod and single-pellet retrieval assays (Crawley, 1999). Open-field testing revealed no significant difference in total distance traveled between *Nex:Cre; MEK1^S217/221E^* and control mice (Fig. 5A, *n*=12, two-tailed unpaired Student's *t*-test, *P*=0.075). The time spent in the center of the open-field arena is a gauge of anxiety-related behavior; however, we did not observe a significant difference (Fig. 5A, *n*=12, two-tailed unpaired Student's *t*-test, *P*=0.21). We also did not observe a significant effect of genotype during accelerating rotarod testing, which assesses motor coordination and balance {Fig. 5B, *n*=15 controls and 14 mutants, two-way repeated measures ANOVA [*F*_(1, 27)_=0.246, *P*=0.624]}. These data reveal that *Nex:Cre; MEK1*^*S217/221E*^ mice have relatively normal global voluntary locomotor behavior, motor coordination and balance.

We next used a single-pellet retrieval task to determine whether there was a deficit in fine forelimb motor function and learning, which relies heavily on the motor cortex (Chen et al., 2014; Kawai et al., 2015; Li and Hollis, 2021). This task assays the ability of mice to reach through a narrow slot with a single forelimb to retrieve a sucrose pellet. We measured the percentage of successful retrievals over the total number of reaching attempts across five consecutive days of testing. Importantly, we found that *Nex:Cre; MEK1^S217/221E^* mutants exhibited a significant decrease in success rate relative to that of controls {Fig. 5C,D, mean±s.e.m., *n*=11 controls and 8 mutants, two-way repeated measures ANOVA, main effect of genotype [*F*_(1,16)_=11.04, *P*=0.004]}. Although no significant difference between genotypes was noted in success rate on the first day of testing [least significant different (LSD) post hoc test, *P*=0.59] or in the number of attempts [*F*_(1,16)_=0.06, *P*=0.81], control mice showed a significant increase in success rate over 5 days of testing (Fig. 5C,D, mean±s.e.m., post hoc LSD two-tailed unpaired *t*-test for day 1 versus day 5, *P=0.016, *n*=11). In contrast, the success rate in mutant mice did not increase over time and was significantly decreased relative to that of controls during the last three days of testing (LSD posthoc test, *P<0.05). Collectively, these results indicate that the *Nex:Cre; MEK1^S217/221E^* mice do not exhibit initial, basal differences in fine forelimb motor function, but display a significant impairment in learning a skilled forelimb single-pellet retrieval task.

### Mouse model for studying layer V autonomous effects of ERK1/2 signaling

As *Nex:Cre* recombines in all cortical layers, it is unclear whether axonal deficits are due to direct layer V-specific effects or changes in upstream layers in the cortical circuit, such as layers II/III*.* To determine the layer V-autonomous functions of ERK1/2 signaling, we used a *Rbp4:Cre* mouse that drives recombination primarily in layer V neurons in the cortex (Gong et al., 2007; Kozorovitskiy et al., 2012) and outside the nervous system in the liver (Steinhoff et al., 2021; Thompson et al., 2017). Inducible deletion of *Erk1/2* in adult mice has been shown to trigger hepatocyte death and increased mortality, whereas ERK1/2 hyperactivation induces hepatocyte proliferation and carcinoma (Cingolani et al., 2022; Huynh et al., 2003; Ito et al., 1998). Attempts to generate *Rbp4:Cre; Erk1^−/−^; Erk2^fl/fl^* loss-of-function mice did not result in viable births (0 of 85, expected 1:4 births). However, *Rbp4:Cre; MEK1^S217/221E^; Ai9^+/−^* mice were viable into adulthood, but exhibited hepatomegaly (Fig. S3M). Nonetheless, *Rbp4:Cre; MEK1^S217/221E^; Ai9^+/−^* mice expressed tdTomato and elevated levels of MEK1 in cortical layer V somas and axon projections and provided a model for evaluating layer V-autonomous effects (Fig. S3A-L).

### Layer V-directed expression of MEK1^S217/221E^ reduces cortical axon arborization

**Fig. 6.**
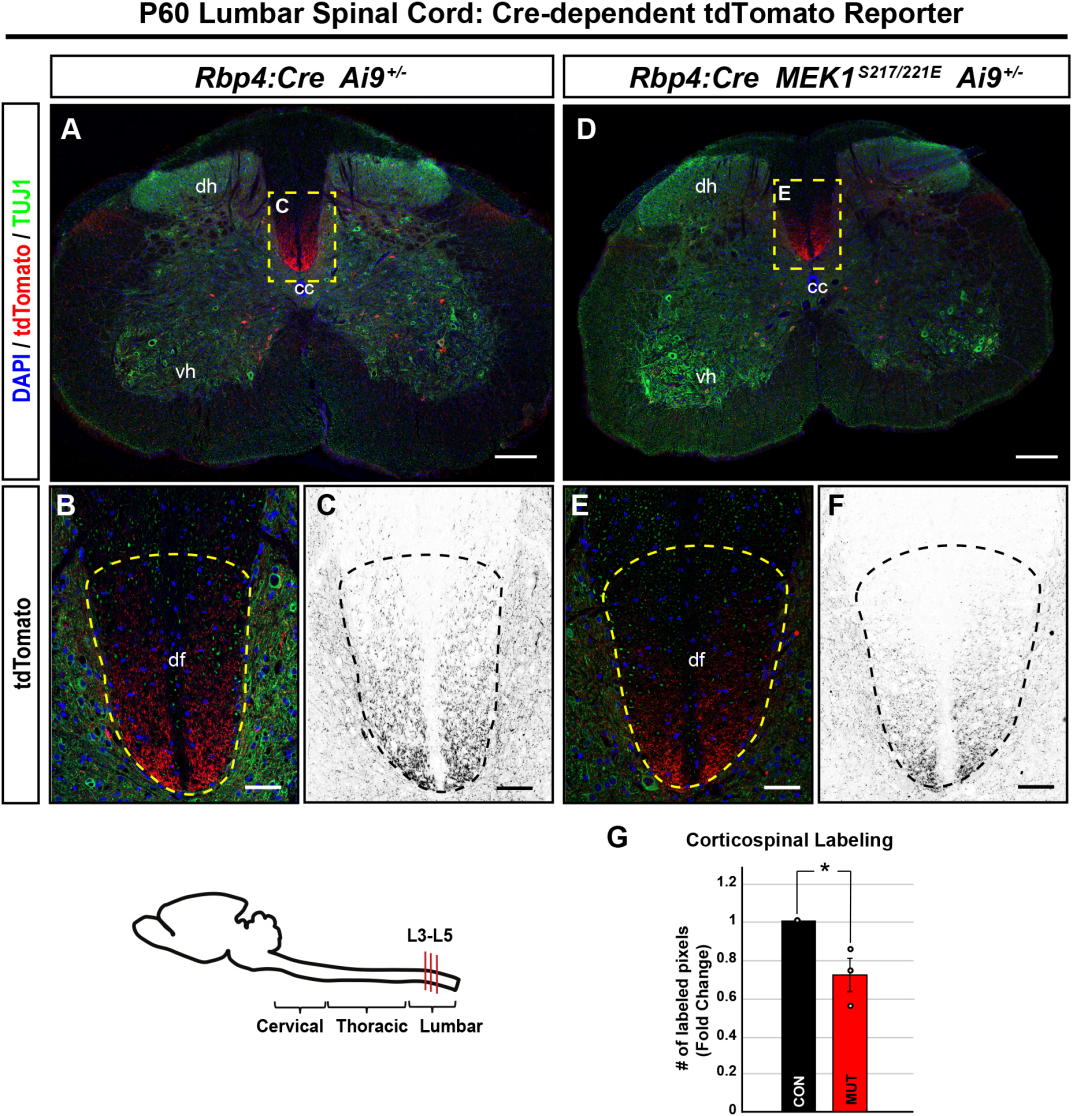
**MEK1^S214/221E^ expression in cortical layer V reduces corticospinal axon elongation in the spinal cord dorsal fasciculus.** (A-G) Sections from *Rbp4:Cre; Ai9^+/−^* control (A-C) and *Rbp4:Cre; MEK1^S17/221E^; Ai9^+/−^* mutant (D-F) mice were labeled for descending cortical layer V-derived corticospinal axons (dashed yellow boxes). Analysis of cross-sectional lumbar segments revealed a statistically significant 28% reduction of tdTomato^+^ labeled pixels in the dorsal funiculus (df) of *Rbp4:Cre; MEK1^S217/221E^; Ai9^+/−^* mice (E,F, dashed yellow outlines) compared to those in *Rbp4:Cre; Ai9^+/−^* controls (B,C; quantification in G, mean±s.e.m., *n*=3, two-tailed unpaired Student's *t*-test, *P<0.05). The schematic shows a midsagittal outline of the mouse central nervous system, and lumbar segments L3-L5 are indicated. CC, central canal; dh, dorsal horn; vh, ventral horn. Scale bars: 200 µm (A,D); 20 µm (B,C,E,F).

We did not observe a decrease in tdTomato^+^ cortical layer V neuron density in M1 of *Rbp4:Cre; MEK1^S217/221E^; Ai9^+/−^* mutants relative to that in controls (control, 372±9 labeled pixels/transduced cell; mutant=477±38  labeled pixels/transduced cell; mean±s.e.m., two-tailed unpaired Student's *t*-test, *P*=0.053), consistent with past findings assessing the proportion of layer V CTIP2^+^ neurons in *Nex:Cre; MEK1^S217/221E^* mice (Xing et al., 2016) (Fig. S3B,E). We next asked whether hyperactive MEK1 expression in layer V is sufficient to reduce axon extension. Layer V-derived subcortical projections in the spinal cord dorsal funiculus were labeled by the Cre-dependent tdTomato/RFP reporter *Ai9^+/−^* in *Rbp4:Cre* mice (Fig. S3G,H,J,K). We observed that *Rbp4:Cre; MEK1^S217/221E^; Ai9^+/−^* mice had a 28±8% reduction in tdTomato^+^ pixel density in the lumbar dorsal funiculus compared to that in controls (Fig. 6A-G) (mean±s.e.m., *n*=3, two-tailed unpaired Student's *t*-test, *P*=0.03).

**Fig. 7.**
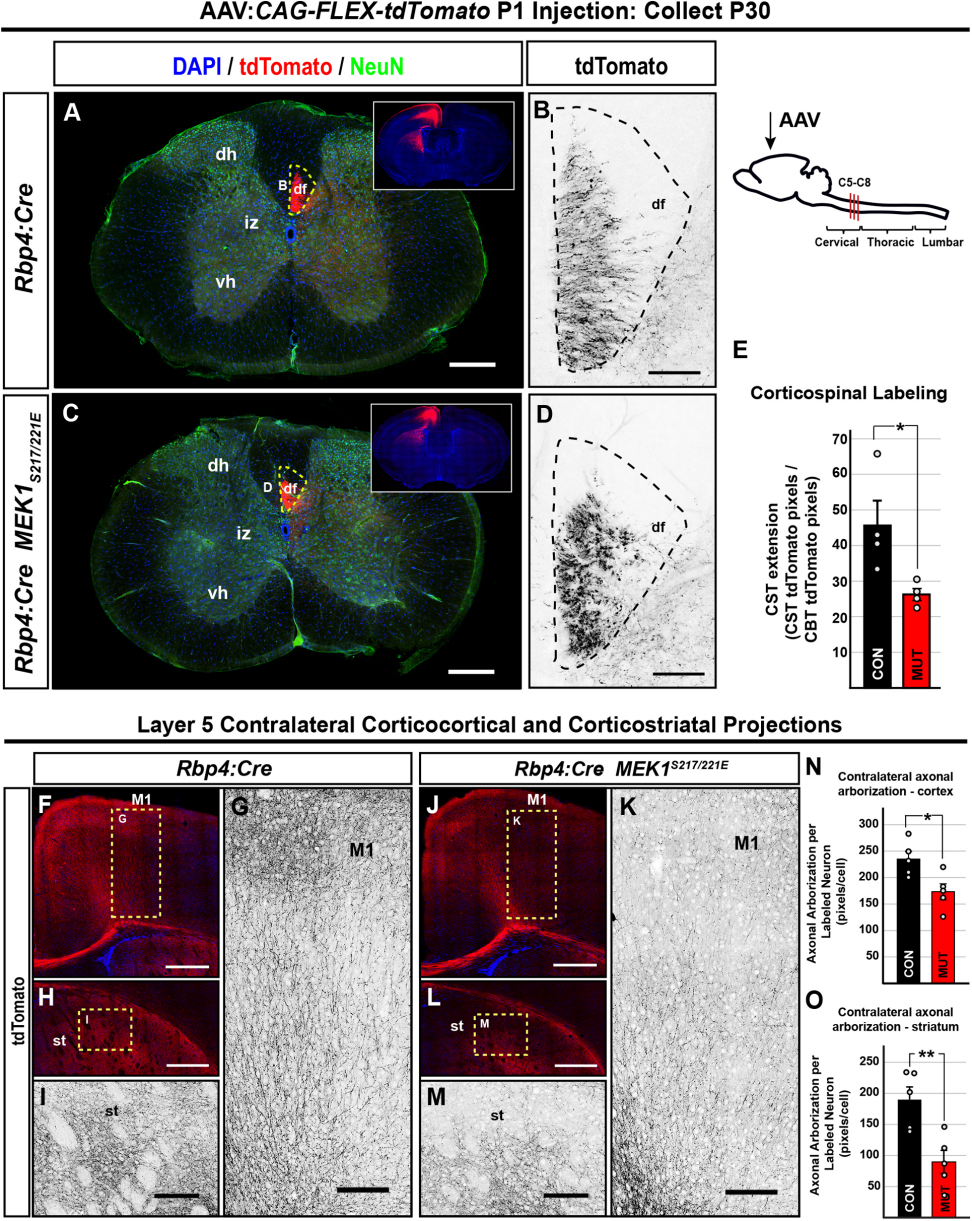
**Corticospinal, corticocortical and corticostriatal layer V projections are reduced in *Rbp4:Cre; MEK1^S217/221E^* mutants.** (A-E) Unilateral AAV labeling of descending corticospinal axons derived from the primary motor cortex (M1) in *Rbp4:Cre* mice (A,C). Cross sections of cervical spinal cord segments reveal a reduction of descending tdTomato^+^ corticospinal axons in the dorsal funiculus of *Rbp4:Cre; MEK1^S217/221E^* mutants (D) compared to those in *Rbp4:Cre* control mice (B) when normalized to corticobulbar tract (CBT) labeling (E, mean±s.e.m., *n*=4, two-tailed unpaired Student's *t*-test, *P=0.036). The schematic shows a midsagittal outline of the mouse central nervous system, and the AAV injection site and cervical segments are indicated. (F-O) Virally labeled *Rbp4:Cre; MEK1^S217/221E^* axons (F,J) had significantly reduced arborization in the contralateral motor cortex (K) compared to that in *Rbp4:Cre* controls (G) when normalized to the number of transduced cells in M1 (quantification in N, mean±s.e.m., *n*=4, two-tailed unpaired Student's *t*-test, *P=0.019). Additionally, *Rbp4:Cre; MEK1^S217/221E^* mice had significantly reduced arborization of the dorsolateral striatum (L,M) compared to that in *Rbp4:Cre* controls (H,I) (quantification in O, mean±s.e.m., *n*=5, two-tailed unpaired Student's *t*-test, **P=0.007). Scale bars: 200 µm (A,C); 20 µm (B,D); 500 µm (F,H,J,L); 150 µm (G,I,K,M). CST, corticospinal tract; df, dorsal funiculus; dh, dorsal horn, iz, intermediate zone, st, striatum; vh, ventral horn.

The widespread bilateral labeling of all cortical layer V neurons in *Rbp4:Cre; Ai9^+/−^* mice made it challenging to determine the anatomical source of axonal labeling. Therefore, we used focal neonatal injection of Cre-dependent tdTomato/RFP adeno-associated virus (AAV) into M1 to unilaterally label neurons at birth. Comparable numbers of neurons were labeled in M1 in control and *Rbp4:Cre; MEK1^S217/221E^* mice at P30 (mean±s.e.m., *n*=5, two-tailed unpaired Student's *t*-test, *P*=0.400) (Fig. S4C). Although there was no difference in tdTomato labeling in the corticobulbar tract, mutants had reduced tdTomato labeling in the cervical dorsal funiculus compared to that in controls, consistent with decreased CST elongation (Fig. 7A-E; Fig. S4D-H). Intracortical arborization in contralateral M1 of *Rbp4:Cre; MEK1^S217/221E^* mice was significantly reduced compared to that in control mice (mean±s.e.m., *n*=5, two-tailed unpaired Student's *t*-test, *P*=0.019) (Fig. 7F,G,J,K, quantification in Fig. 7N). Contralateral dorsolateral striatal arborization was also significantly reduced in mutants compared to that in controls (mean±s.e.m., *n*=5, two-tailed unpaired Student's *t*-test, *P*=0.007) (Fig. 7H,I,L,M, quantification in Fig. 7O). Collectively, these results indicate that hyperactive ERK1/2 signaling in cortical glutamatergic layer V neurons is sufficient to reduce axonal elongation of multiple projection neurons.

### Reduction in the cortical wiring of layer V is expendable for skilled motor learning

**Fig. 8.**
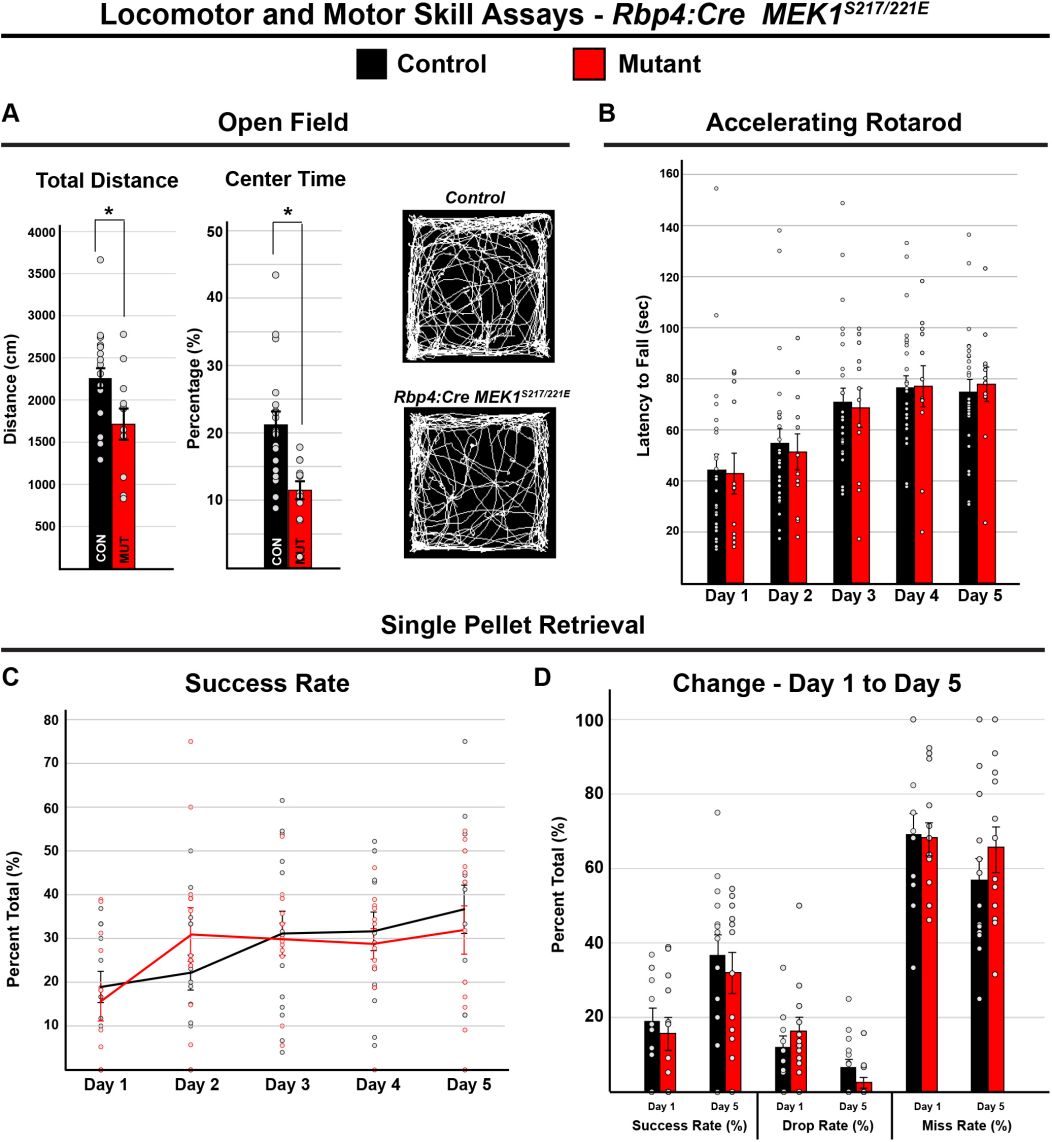
**MEK1^S217/221E^ in cortical layer V glutamatergic neurons does not disrupt motor learning.** (A) Open-field testing of *Rbp4:Cre; MEK1^S217/221E^* and control mice revealed that mutants exhibited a significant reduction in total distance traveled (mean±s.e.m., *n*= 22 controls and 11 mutants, two-tailed unpaired Student's *t*-test, *P=0.01) and percentage of time spent in the center of box (mean±s.e.m., *n*=22 controls and 11 mutants, two-tailed unpaired Student's *t*-test, *P=0.001). (B) Accelerating rotarod testing revealed no significant effect of genotype over 5 days of testing control and mutant mice {mean±s.e.m., *n*=26 controls and 12 mutants, [*F*_(1,36)_=0.007, *P*=0.933]}. (C,D) In the single-pellet retrieval task, there was no significant effect of genotype on daily success rate {C, mean±s.e.m., *n*=14 controls and 12 mutants, two-way repeated-measures ANOVA [*F*_(1,24)_=0.03, *P*=0.88]}. Both mutants and controls improved their success rate over 5 days {D, mean±s.e.m., *n*=14 controls and 12 mutants, two-way repeated-measures ANOVA [*F*_(4,24)_=5.81, *P*<0.01]}.

As *Rbp4:Cre; MEK1^S217/221E^* mice exhibited reduced axonal extension of cortical projection neurons, we tested whether these mutants display deficits in motor behavior. Hepatomegaly was observed in *Rbp4:Cre; MEK1^S217/221E^* mice, and mouse models of chronic liver disease have been shown to exhibit hypo-locomotion in the open-field test, but they show little change in motor learning or anxiety-like phenotypes (Jover et al., 2006). Compared to control mice, *Rbp4:Cre; MEK1*^*S217/221E*^ mutants showed a significant decrease in voluntary distance traveled in the open-field assay (*n*=22 controls and 11 mutants, two-tailed unpaired Student's *t*-test, *P*=0.015) and a decrease in the proportion of time spent in the center (two-tailed unpaired Student's *t*-test, *P*=0.001) (Fig. 8A). In contrast, no significant effect of genotype was detected on the accelerating rotarod task {*n*=26 controls and 12 mutants, two-way repeated measures ANOVA [*F*_(1,36)_=0.007, *P*=0.933]} (Fig. 8B).

In the single-pellet retrieval task, we found that mild food restriction in *Rbp4:Cre; MEK1^S217/221E^* mice resulted in health issues of unknown origin and spontaneous lethality. Thus, we substantially reduced the period of food restriction relative to that in our *Nex:Cre* experiments and only analyzed reaching and grasping behaviors during the first 5 min of each trial when mice were highly motivated. In stark contrast to *Nex:Cre; MEK1^S217/221E^* mutants, we found no significant effect of genotype on the success rate between control and *Rbp4:Cre; MEK1^S217/221E^* mice {*n*=14 controls and 12 mutants, two-way repeated measures ANOVA [*F*_(1,24)_=0.03, *P*=0.88]} (Fig. 8C-D). Taken together, these results indicate that layer V-specific hyperactivation of MEK1 in glutamatergic neurons is insufficient to disrupt motor skilled learning.

## DISCUSSION

Resolving the cell-specific effects of pathological ERK1/2 activity may help inform the rational design of therapeutic approaches for multiple neurodevelopmental conditions. Here, we tested whether cortical excitatory neuron-directed expression of hyperactive MEK1^S217/221E^ during early development modulates long-range axon outgrowth from sensorimotor cortices, expression of ARC and motor-related behaviors. MEK1 hyperactivation selectively mimicked a core downstream feature of multiple RASopathies without direct modulation of multiple RTK-linked signaling cascades associated with upstream mutations. We generated mice expressing Cre-dependent MEK1^S217/221E^ and *Nex:Cre* to target cortical excitatory neurons, but not cortical GABAergic neurons or glia. Axonal levels of pERK1/2 were persistently increased in these mutants. Importantly, ERK1/2 hyperactivation coincided with reductions in corticocortical, corticostriatal and corticospinal axon projections arising from motor cortices. We also observed lower expression of the activity-dependent protein ARC within the mature cortex of mutant mice. Although many basic motor behaviors remained intact, skilled motor learning in a reaching task was inhibited in these mutants. Lastly, we demonstrate that a layer V-specific MEK1^S217/221E^ mutant mouse recapitulated axonal outgrowth deficits, but motor learning was not altered. Overall, our data show that layer V-autonomous expression of MEK1^S217/221E^ disrupts the development of long-range cortical axon projections, whereas MEK1 hyperactivation in developing *Nex:Cre*-expressing neurons appears to be sufficient to disrupt specific aspects of skilled motor learning (Fig. S5).

### Compartment-specific ERK1/2 activation in *MEK1^S217/221E^*-expressing neurons

*In vitro* studies have been vital for quantitative studies of kinase signaling and pharmacological inhibitors, but the precise effect of RASopathy variants on pERK1/2 levels and distribution are more challenging to measure *in vivo*. We generated mutants with increased hyperactive MEK1 levels in developing excitatory neurons and observed increased levels of pERK1/2 in axonal, but not somal and nuclear, compartments. Recent studies have shown that callosal layer II/III projections have enriched ERK2 protein within axonal growth cones (Engmann et al., 2022; Poulopoulos et al., 2019; Shigeoka et al., 2016). It will be important to explore whether enrichment of ERK1/2 pathway components within the axon renders this compartment relatively vulnerable to RASopathic variants. High-resolution measurement of RASopathic kinase activation in distinct subcellular domains *in vivo* may further illuminate core pathological mechanisms.

Paradoxically, the somal and nuclear levels of pERK1/2 were reduced in adult mutant excitatory neurons. A similar phenomenon has been observed in *Drosophila* MEK1 mutants, possibly due to early changes in the expression of morphogens during development (Goyal et al., 2017). The compensatory mechanisms that appear to override the biochemical hyperactivity of the MEK1^S217/221E^ mutant in neuronal soma and nuclei may involve ERK1/2-directed phosphatases enriched within somal compartments or developmental changes in upstream ERK1/2 activators (Caunt et al., 2008; Jeffrey et al., 2007; Li et al., 2007; Lim et al., 2015; Parmar et al., 2015; Poulopoulos et al., 2019). Nevertheless, pharmacological inhibitors of the ERK1/2 signaling cascade are actively being explored for clinical use in RASopathies (Guilding et al., 2007; Hatzivassiliou et al., 2010; Morris et al., 2013; Nakamura et al., 2009; Zamorano et al., 2018). For example, treatment with MEK1/2 inhibitors during postnatal stages reverses cardiac deficits and tumorigenesis in RASopathy models and patients with NS (Andelfinger et al., 2019; Hilal et al., 2023; Wu et al., 2011). However, if developmental compensatory mechanisms lead to paradoxically reduced levels of pERK1/2 in select neuronal compartments, it is unclear whether pharmacological inhibition would be beneficial. Additional quantitative analyses of compartment-specific changes in pERK1/2 levels in RASopathy models may help inform the use of systemic pharmacological inhibitors.

### MEK-ERK1/2 hyperactivity reduces cortical long-range axon arborization

MRI-based studies of individuals with NF1 and NS have discovered abnormalities in both functional and structural connectivity (Fattah et al., 2021; Filippi et al., 2013; Rai et al., 2023). Although oligodendrocyte-mediated changes in the myelination of axons are likely involved, the neuron-autonomous contributions to aberrant connectivity are less understood. We show that neuron-specific MEK1^S217/221E^-expressing mice did not exhibit significant loss of cortical layer V neurons, but layer V-autonomous hyperactivation of ERK1/2 reduced the arborization of corticocortical, corticostriatal and corticospinal axons. *In vitro* studies have demonstrated that ERK1/2 is necessary for aspects of trophic factor-induced axon outgrowth (Markus et al., 2002; Ozdinler and Macklis, 2006), axon guidance (Bagnard et al., 2004; Forcet et al., 2002; Yun et al., 2005), cytoskeletal organization (Atwal et al., 2003; Goold and Gordon-Weeks, 2005; Ray and Sturgill, 1987) and local protein translation in growth cones (Campbell and Holt, 2003). Whether hyperactive ERK1/2 activity disrupts these molecular processes or modulates other intracellular events associated with axon outgrowth *in vivo* is unclear. Axonal outgrowth in humans occurs during gestational stages, which makes therapeutic intervention more difficult. RASopathies are often diagnosed within the first years of life (Zenker et al., 2022). Therefore, it may be beneficial to study postnatal stages of axonal refinement and pruning in RASopathies, which may be more accessible to current treatment strategies.

Animal models of upstream RASopathic variants in the *NF1*, *PTPN11* and RAS genes have observed changes in multiple RTK-linked downstream intracellular cascades, including PI3K/Akt, JAK/STAT, PKC, mTOR and ERK1/2, and inhibitors of multiple cascades are capable of reversing certain cellular deficits (Anastasaki and Gutmann, 2014; Brown et al., 2012; Langdon et al., 2012; López-Juárez et al., 2017; Titus et al., 2017). Our work now shows that downstream MEK-ERK1/2 hyperactivity is sufficient to reduce long-range axon arborization *in vivo*. Understanding whether upstream mutations also drive neuron-autonomous deficits in long-range excitatory neuron axonal outgrowth may help inform the design of personalized, mutation-specific approaches to RASopathic therapeutics.

### *Nex:Cre; MEK1^S217/221E^* mice exhibit decreased *Arc* expression and deficits in skilled motor learning

*Arc* is an immediate early gene that rapidly responds to neuronal activity and regulates synaptic plasticity (Steward and Worley, 2001; Steward et al., 1998). *Arc* has been shown to be dysregulated in fragile X and Angelman syndrome (Greer et al., 2010; Zalfa et al., 2003); however, whether individuals with RASopathies have aberrant *ARC* expression is not clear. Several reports show that glutamate and BDNF drive *Arc* transcription and translation via ERK1/2 (Chotiner et al., 2010; Correa et al., 2012; Nikolaienko et al., 2017; Pintchovski et al., 2009; Rao et al., 2006; Ying et al., 2002). Notably, our results indicate that MEK1 hyperactivation during early neuronal development unpredictably resulted in reduced pERK1/2 levels in the soma and nucleus of adult neurons. It is perhaps not surprising that the ARC protein is also decreased in adult mutant cortices, in agreement with our past report showing that P21 *Nex:Cre; MEK1^S217/221E^* mice have reduced levels of *Arc* mRNA (Xing et al., 2016). Hippocampal excitatory neurons in a developing forebrain-directed RASopathy mutant, *Emx1:Cre Ptpn11^D61Y^*, exhibit analogous reductions in depolarization-induced pERK1/2 and BDNF promoter activity (Altmüller et al., 2017). We propose that the reduced levels of pERK1/2-ARC in cortical neuron soma and nuclei may be due to decreases in the development of cortical glutamatergic synapses or excitatory activity as a consequence of diminished long-range axon outgrowth.

In line with the reduced expression of *Arc*, our data show that *MEK1^S217/221E^* expression in *Nex:Cre* mice is sufficient to disrupt skilled motor learning in a single-pellet reaching task. It will be important to explore this broader deficit in future studies with other forms of procedural learning and cognitive behaviors. *Nex* (also known as *Neurod6*) predominantly drives Cre expression in immature cortical excitatory neurons. We cannot exclude that other *Nex-*expressing neuronal populations could contribute to this behavioral phenotype (Goebbels et al., 2006). Interestingly, restricting MEK1^S217/221E^ to layer V with *Rbp4:Cre* does not diminish skilled motor learning. Motor learning is known to involve a complex, distributed set of neuronal populations and the mixed genetic backgrounds of mice used in this study may have reduced our ability to detect more subtle changes in behavior. Nonetheless, *MEK1^S217/221E^* mutants expressing either *Nex:Cre* or *Rbp4:Cre* exhibited layer V axon outgrowth deficits. It seems likely that functional deficits in the larger repertoire of cortical neurons targeted in *Nex:Cre; MEK1^S217/221E^* relative to *Rbp4:Cre* strains could contribute to the learning defect we observed. For example, layer II/III neurons in the motor cortex have been shown to be activated in a skilled reaching task and contain a sparse population of pyramidal neurons with some of the highest levels of pERK1/2 in the normal adult mouse cortex (Levy et al., 2020). We found a reduction in the number of layer II/III neurons with high levels of pERK1/2 in the motor cortex of *Nex:Cre; MEK1^S217/221E^* mice, which are not directly targeted in *Rbp4:Cre* strains. Additional research will be needed to explore the normal functions and development of high pERK1/2-expressing layer II/III neurons and whether they are particularly vulnerable to RASopathic signaling.

## MATERIALS AND METHODS

### Transgenic mice

All animal experiments were performed in accordance with established procedures approved by the Institutional Animal Care and Use Committee of Arizona State University (protocol #22-1924R) and National Institutes of Health guidelines for the use and care of laboratory animals. Mice used in this work were housed in standard conditions and kept on a 12-h/12-h light/dark cycle with *ad libitum* access to food and water unless indicated otherwise.

*Nex:Cre* [*Neurod6^tm1(cre)Kan^*] mice were kindly provided by Dr Klaus Nave and Dr Sandra Goebbels (Max Planck Institute for Multidisciplinary Sciences, Göttingen, Germany) (MGI:2668659) (Goebbels et al., 2006). *Rbp4:Cre* mice were obtained from the Mutant Mouse Resource and Research Centers (RRID:MMRRC_031125-UCD) (Gong et al., 2007). *MEK1^S217/221E^* [Tg(CAG-cat,-Map2k1*)*243Rbns*] mice were generated and generously provided by Dr Maike Krenz and Dr Jeffrey Robbins (Cincinnati Children's Hospital Medical Center, Cincinnati, OH, USA) (MGI:3822131) using a *MEK1^S217/221E^* construct derived from site-directed mutagenesis of rabbit MEK1 as described in Alessi et al. (1994) (Krenz et al., 2008). Cre-dependent *tdTomato/Ai9* [B6.Gt(ROSA)26Sor^tom(CAG-tdTomato)Hze^/J] mice were ordered from The Jackson Laboratory (RRID:IMSR_JAX:007909) (Madisen et al., 2010). The double and triple transgenic mice used in this study were on mixed genetic backgrounds. For experiments that relied on the Cre-dependent tdTomato/RFP reporters, all ‘controls’ were Cre^+^. Littermates that were either wild-type mice, Cre-negative mice or Cre-expressing mice lacking *MEK1^S217/221E^* were pooled as ‘controls’ for the remaining experiments unless stated otherwise. Each experiment was replicated a minimum of three times from independent litters.

Genomic DNA was extracted from toe or tail samples and amplified with standard polymerase chain reaction techniques. Primers for gene amplification are as follows: for Cre, 5′-GCTAAACATGCTTCATCGTCGG-3′ and 5′-GATCTCCGGTATTGAAACTCCAGC-3′ amplify a 645 bp allele; for *MEK1^S217/221E^*, 5′-GTACCAGCTCGGCGGAGACCAA-3′ and 5′-TTGATCACAGCAATGCTAACTTTC-3′ amplify a 600 bp allele; and for *Ai9*, 5′-CTGTTCCTGTACGGCATGG-3′ and 5′-GGCATTAAAGCAGCGTATCC-3′ amplify a 196 bp allele.

### Tissue preparation

Transcardial perfusions were performed on mice using a 4% paraformaldehyde/PBS solution and dissected brains were post-fixed overnight at 4°C. Brains were then cryopreserved via serial incubations in 15% and 30% sucrose/PBS solutions prior to embedding in OCT (Tissue-Tek, 4583) and rapid freezing. Cryostat sections were collected in ice-cold PBS for free-floating staining or mounted on Fisherbrand Superfrost Plus slides (Thermo Fisher Scientific, 12-550-15) and air dried prior to staining. For material sectioned using a vibratome (Electron Microscopy Sciences, 7000smz-2), fixed adult brains were mounted in agarose and sections were collected in PBS prior to free-floating immunohistochemistry.

### Viral injections and image quantification

P1 pups were removed from their home cage as a group and individually cryo-anesthetized on ice for 4-6 min. Viral injections were then performed immediately with 50 nl of solution using a 32-gauge beveled needle fitted to a Hamilton 5 µl Neuros syringe mounted on a manipulator arm. After injections, pups were placed on a 37°C heated surface for recovery and returned to their home cage as a group. Adeno-associated viral vectors containing a Cre-dependent tdTomato construct under the control of a strong CAG promoter [AAV:*CAG-FLEX-tdTomato*, University of North Carolina (UNC) Viral Vector Core] were used for viral tracing experiments. For quantification of virally labeled tdTomato^+^ axonal arborization in the forebrain, we first manually assessed the number of tdTomato^+^ neurons in M1 from confocal images of at least three sections near the center of the injection site. Confocal images of axonal target domains from at least three sections were automatically thresholded in ImageJ using the Otsu method (Otsu, 1979). The total number of tdTomato^+^ pixels within the target domain was normalized to the number of labeled M1 neurons within the injection site to provide a relative estimate of axonal arborization. To quantify axon elongation along the spinal cord, tdTomato^+^ immunolabeling in the cervical dorsal funiculus was normalized to immunolabeling in the hindbrain corticobulbar tract. Representative images were cropped, adjusted for brightness and contrast, and inverted in Photoshop for clear visualization of axonal projections.

### Immunohistochemistry

Tissue sections were rinsed with PBS containing 0.1% Triton X-100 (PBST) and blocked with 5% normal donkey serum (NDS) in PBST at room temperature for ∼1 h. Primary antibodies were diluted in PBST containing 5% NDS and incubated overnight at 4°C with gentle agitation. The primary antibodies used were: rabbit anti-MEK1 (RRID:AB776273, Abcam, ab32091, 1:1000), rabbit anti-phospho-p44/42 MAPK (ERK1/2) (Thr202/Tyr204) (RRID:AB_2315112, Cell Signaling Technologies, 4370, 1:750), rabbit anti-ARC (RRID:AB_887694, Synaptic Systems, 156-003, 1:750), mouse anti-β3 tubulin (TUJ1, RRID:AB_10063408, BioLegend, 801202, 1:1000), rabbit anti-RFP/tdTomato (RRID:AB_2209751, Rockland Immunochemicals, 600-401-379, 1:1000), chicken anti-RFP/tdTomato (RRID:AB_10704808, Rockland Immunochemicals, 600-901-379, 1:1000) and mouse anti-NeuN (RRID:AB_2298772, Millipore Sigma, MAB377, 1:1000). After rinsing in PBS three times, secondary antibodies diluted in PBST containing 5% NDS with DAPI (Sigma-Aldrich, 10236276001) were added and incubated overnight at 4°C with gentle agitation. The secondary antibodies include Alexa Fluor 488, 546, 568 and 647 conjugated to anti-rabbit IgG, anti-mouse IgG or anti-chicken IgY (Invitrogen, A31573, A21202, A10042, A11041 and A11071). Imaging was performed using a Zeiss LSM 800 or Leica SP5 laser scanning confocal microscope.

### Confocal image analysis and quantification

The number of pERK1/2-labeled cells in M1 layers II/III were counted from confocal images collected with a 20× objective in at least two hemi-sections per mouse and averaged. We quantified the level of pERK1/2 immunolabeling in high-resolution confocal images of layer V neurons collected with a 63×1.4 NA objective using the same acquisition settings. Individual NeuN^+^ pyramidal-shaped somas that were >100 µm^2^ in area with a clear DAPI-labeled nucleus in cortical layer V of the motor cortex were randomly selected by an observer unaware of sample identities. The integrated density of pERK1/2 immunolabeling in the cytoplasm and nucleus was then measured. A total of 68 control and 83 mutant neurons was analyzed from at least three sections per mouse, four mice per genotype, representing a total sampling area >14,000 µm^2^ per genotype.

To estimate the levels of pERK1/2 immunoreactivity in TUJ1^+^ callosal axons, standard confocal images of the corpus collosum were collected and analyzed. Standard confocal images of at least two tissue sections of the corpus collosum per mouse were collected and automatically thresholded in ImageJ in an unbiased manner using the default settings of Moments Autothreshold (Tsai, 1985). Colocalized pERK1/2^+^/TUJ1^+^ pixels were determined using Pierre Bourdoncle's Colocalization plugin (https://imagej.net/ij/plugins/colocalization.html) with default settings and normalized to the total number of TUJ1^+^ pixels. Representative images were subsequently acquired in Airyscan mode using optimized settings to visualize the localization of pERK1/2 within TUJ1^+^ axons.

To quantify CST elongation in *Rbp4:Cre; Ai9^+/−^* mice, confocal images of the dorsal funiculus in at least three lumbar segments per control and *MEK1^S217/221E^*-expressing mice were binarized and the relative change in the number of tdTomato^+^ pixels was determined. Representative images have been cropped and adjusted for brightness and contrast in Photoshop for presentation.

### Western blotting

Dorsal cortices were lysed in RIPA buffer (0.05 M Tris-HCl, pH 7.4, 0.5 M NaCl, 0.25% deoxycholic acid, 1% Triton X-100 and 1 mM EDTA, Millipore) supplemented with 0.1% SDS, protease inhibitor (Sigma-Aldrich, P2714) and phosphatase inhibitor cocktail II and III (Sigma-Aldrich, P5726) via sonication. Lysates were then cleared by centrifugation and protein concentrations determined by Bradford Assay using a Peirce Protein Assay Kit (Thermo Fisher Scientific, 23200). Equal amounts of protein were denatured in reducing sample buffer, separated by SDS-PAGE gels (Bio-Rad, 456-1035 and 456-8095) and transferred to PVDF membranes (Bio-Rad, 162-0177). Blots were blocked with 5% low-fat milk in TBS containing 0.1% Tween 20 (TBST) for 1 h at room temperature, then incubated overnight at 4°C with primary antibodies in 4% bovine serum albumin in TBST. The primary antibodies used were: rabbit anti-MEK1 (RRID:AB_776273, Abcam, ab32091, 1:1000), rabbit anti-phospho-p44/42 MAPK (ERK1/2) (Thr202/Tyr204) (RRID:AB_2315112, Cell Signaling Technologies, 4370, 1:1000), rabbit anti-ERK2 (RRID:AB_732210, Abcam, ab32081, 1:1000), rabbit anti-ARC (RRID:AB_887694, Synaptic Systems, 156-003, 1:1000), rabbit anti-GAPDH (RRID:AB_561053, Cell Signaling Technologies, 2118, 1:1000) and rabbit anti-β-actin (RRID:AB_10950489, Cell Signaling Technologies, 8457, 1:1000). After washing with TBST, the membranes were incubated with donkey anti-rabbit HRP-conjugated secondary antibodies (Jackson Immunoresearch, 711-035-152, 1:2000-1:5000) in 5% milk in TBST for 2 h at room temperature. The membranes were washed with TBST and signals were detected with SuperSignal West Pico chemiluminescent reagent (Thermo Fisher Scientific, 32106) and exposed to radiographic films (Thermo Fisher Scientific, 34089). Quantification of western blots was performed using a high-resolution scanned image of the exposed radiographic film in a grayscale TIFF file format. Bands were selected for quantification of integrated density in ImageJ following background subtraction from an unlabeled region and normalized to the intensity of either the loading control or the non-phosphorylated target.

### Open-field, accelerating rotarod and single-pellet retrieval tests

Open-field testing was performed in an open-top opaque plexiglass box measuring 38×38×30 cm. The area within the box was illuminated by a centered, overhead 40 W spotlight. Video recordings of open-field behavior were scored by an observer who was not aware of the mouse genotypes. Rotarod analysis was performed on an accelerating rotarod (San Diego Instruments). Animals were tested for three trials per day for five consecutive days, accelerating the rotarod from 4 to 40 rpm with a 15 min intertrial interval. The latency to fall was measured for each of three trials during a day and averaged.

A single-pellet retrieval task was performed using only male mice that were acclimated to sucrose pellets (TestDiet, 1811555) in their home cages for at least 1 week prior to testing. Mice were habituated for 10-15 min per day, for 2 days in the testing room and chamber, which consisted of a 15×10×20 cm transparent acrylic box with a 5 mm wide slit on the right and left side of one end that opened to a 10 mm-high food platform. During the subsequent two training days, mice were observed while single sucrose pellets were made regularly available during a 10-20 min session. Only mice that attempted at least 20 reaches were further analyzed.

Daily testing was performed for 10 min per day, for 5 days by placing a single pellet on the food platform. To enhance motivation for obtaining sucrose pellets, *Nex:Cre; MEK1^S217/221E^* mice were mildly food restricted by limiting free feeding to 2-6 h daily while maintaining at least 90% of their initial weight. Due to unexpected negative health effects of mild food restriction in *Rbp4:Cre; MEK1^S217/221E^* mice, these mutants and associated control mice were only restricted during a 2-4 h period prior to a single 5 min session per day. No significant difference was noted in aspects of performance in littermates that were either wild-type mice, Cre-negative mice or Cre-expressing mice lacking MEK1. These mice were therefore pooled as ‘controls’. Attempts were scored as a ‘success’ if the mouse was able to retrieve the sucrose pellet with its preferred paw and place it in its mouth. If the mouse grasped the pellet but failed to return it to its mouth, the attempt was scored as a ‘drop’. If the mouse clearly reached for the pellet but failed to grasp it, the attempt was categorized as a ‘miss’. For each mouse, the proportion of successful out of total attempts was calculated. Accelerating rotarod and single-pellet retrieval results were analyzed using a two-way repeated measures ANOVA in SPSS software.

## Supplementary Material

10.1242/dmm.050570_sup1 Supplementary information
