# Supplementary material for: Hyperactivation of MEK1 in cortical glutamatergic neurons results in projection axon deficits and aberrant motor learning
Source: Dis Model Mech. 2024 Jul 2;17(6):dmm050570. doi: 10.1242/dmm.050570 (PMC11247507; doi:10.1242/dmm.050570)
Supplement: Supplementary information [file dmm-17-050570-s1.pdf]

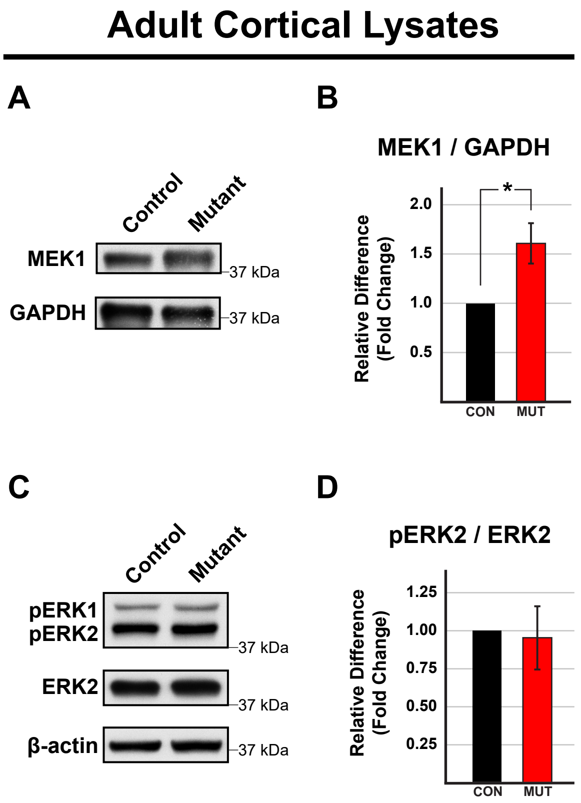

Fig. S1. Increased levels of MEK1, but not pERK1/2, in adult *Nex:Cre; MEK1*$^{S217/221E}$ whole cortical lysates.

A-D. Western blots of adult cortical lysates revealed a 1.61 ± 0.20 fold increase in MEK1 in *Nex:Cre; MEK1*$^{S217/221E}$ mutants when compared to control mice (mean ± SEM, n=3, * Student's t-test p=0.041) (A-B). No significant difference in pERK1/2 levels between mutant and control mice was detected in adult cortical lysates (mean ± SEM, n=3, * Student's t-test p=0.389) (C-D).

Disease Models & Mechanisms • Supplementary information

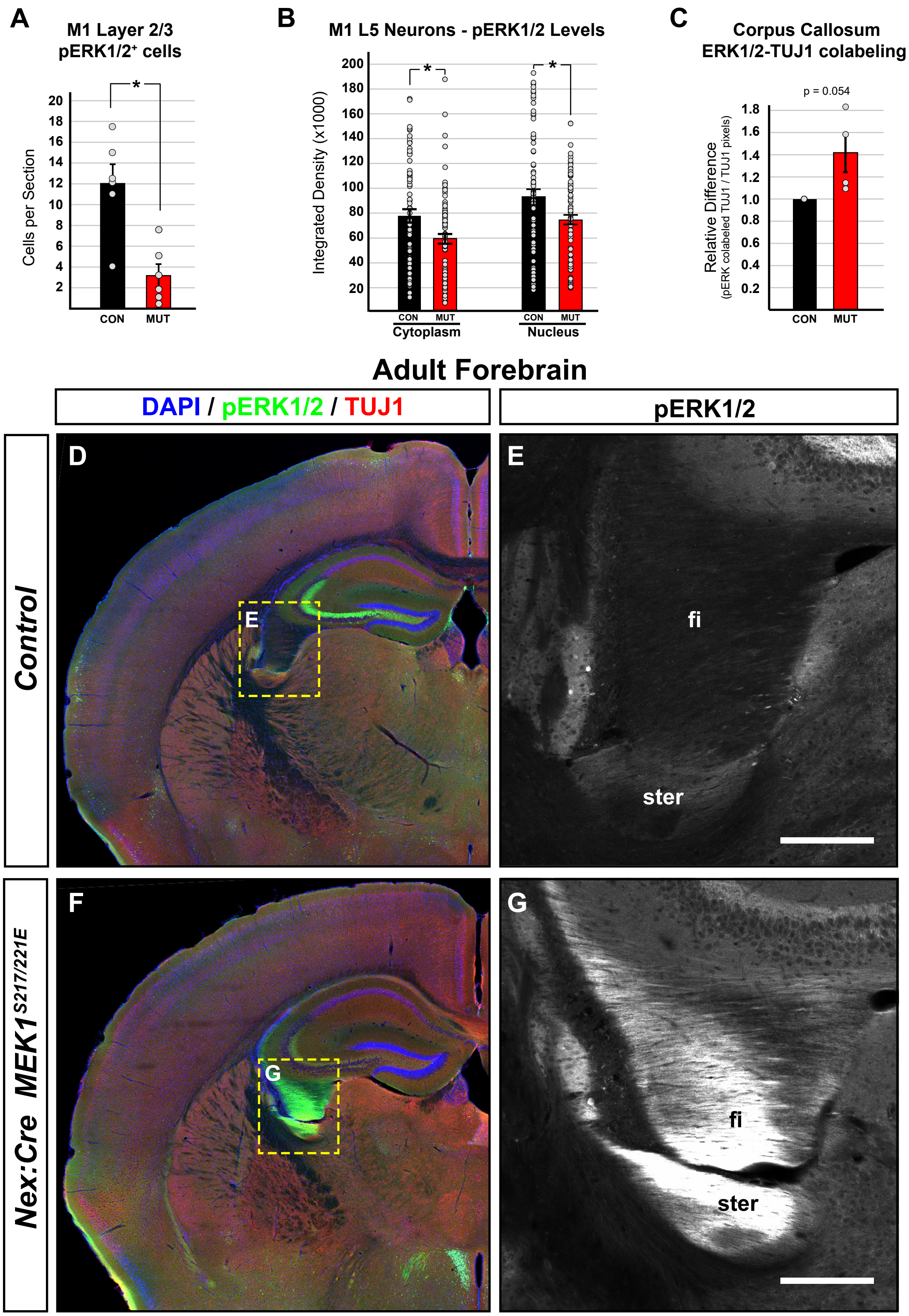
A
M1 Layer 2/3
pERK1/2+ cells
Cells per Section
CON
MUT
B
M1 L5 Neurons - pERK1/2 Levels
Integrated Density (x1000)
Cytoplasm
Nucleus
C
Corpus Callosum
ERK1/2-TUJ1 colabeling
p = 0.054
Relative Difference
(pERK colabeled TUJ1 / TUJ1 pixels)
Adult Forebrain
DAPI / pERK1/2 / TUJ1
pERK1/2
Control
Nex:Cre MEK1 S217/221E
D
E
F
G
fi
ster

Fig. S2. pERK1/2 levels in adult cortical neurons and white matter tracts in mutant mice.

A-C. (A) Quantification of the significant reduction in the number pERK1/2 labeled neurons per section in mutant layer II/III when compared to controls (mean ± SEM, n=6, * Student's t test p=0.002). (B) Analysis of NeuN$^+$ pyramidal neurons in M1 layer V revealed a decrease in cytoplasmic and nuclear levels of pERK1/2 levels when compared to controls (mean ± SEM, n=68 control and 83 mutant neurons from four mice per group, * Student's t-test p<0.01). (C) The increase in pERK1/2 co-localization with TUJ1 in the corpus callosum of mutants was quantified and compared to controls (mean ± SEM, n=4, Student's t test p = 0.054).

D-G. Immunolabeling of forebrain sections at the level of the adult dorsal hippocampus showed a prominent increase of pERK1/2 within the mutant fimbria and stria terminalis (C-D, integrated density (mean gray value x mm^2) in D = 20.4) relative to controls (A-B, integrated density in B = 4.7). These tracts are enriched in axons derived from excitatory neurons in hippocampal CA1-3 and the amygdala, respectively (n=4).

Scale bars = 200µm.

Abbreviations: fi = fimbria, ster = stria terminalis

Disease Models & Mechanisms • Supplementary information

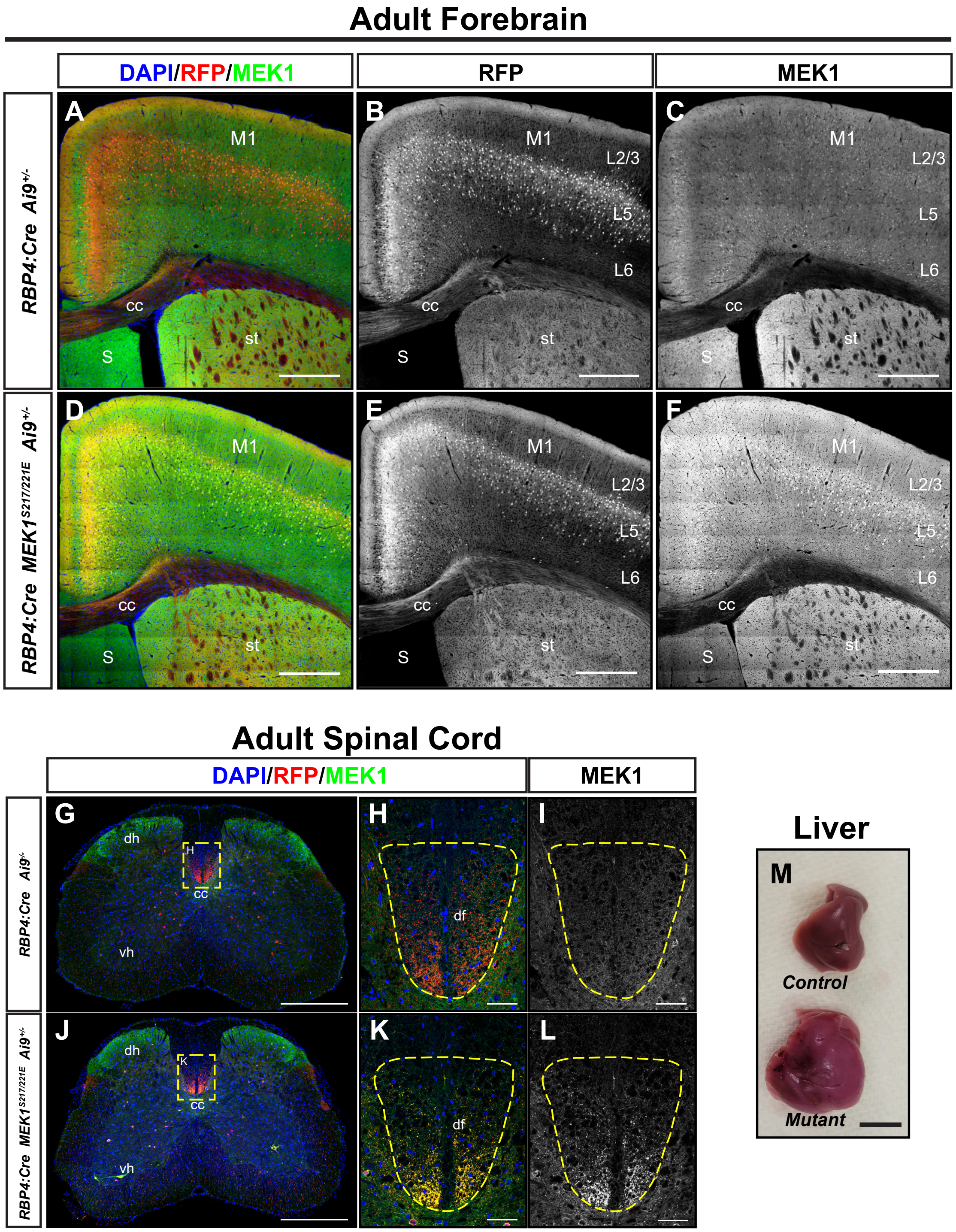

Disease Models & Mechanisms • Supplementary information

Fig. S3. Conditional expression of *MEK1*$^{S217/221E}$ in *RBP4:Cre* mice.

A-F. Representative confocal images of *RBP4:Cre; Ai9*$^{+/-}$ cortices. Both *RBP4:Cre; Ai9*$^{+/-}$ mice control and *RBP4:Cre; MEK1*$^{S217/221E}$*; Ai9*$^{+/-}$ mutants express tdTomato in cortical layer V (A-B, D-E). Immunolabeling of MEK1 showed a robust increase of expression restricted to cortical layer V in mutants (F) compared to controls (C).

G-L. Representative images of cross-sectional lumbar segments showing that tdTomat is detected in descending projections in the dorsal funiculus of controls (G-H) and mutants (J-K). High resolution images reveal increased MEK1 expression in tdTomato-labeled axons of mutants (K-L) compared to controls (H-I).

M. Gross dissection of the mutant liver was suggestive of hepatomegaly when compared to littermate controls (M).

Scale bars: A-F = 500 µm; G, K = 500µm; H-I, K-L = 50µm; M = 1cm.

Abbreviations: M1 = primary motor cortex, cc = corpus collosum, st = striatum, S = septum, dh = dorsal horn, vh = ventral horn, cc = central canal, df = dorsal funiculus

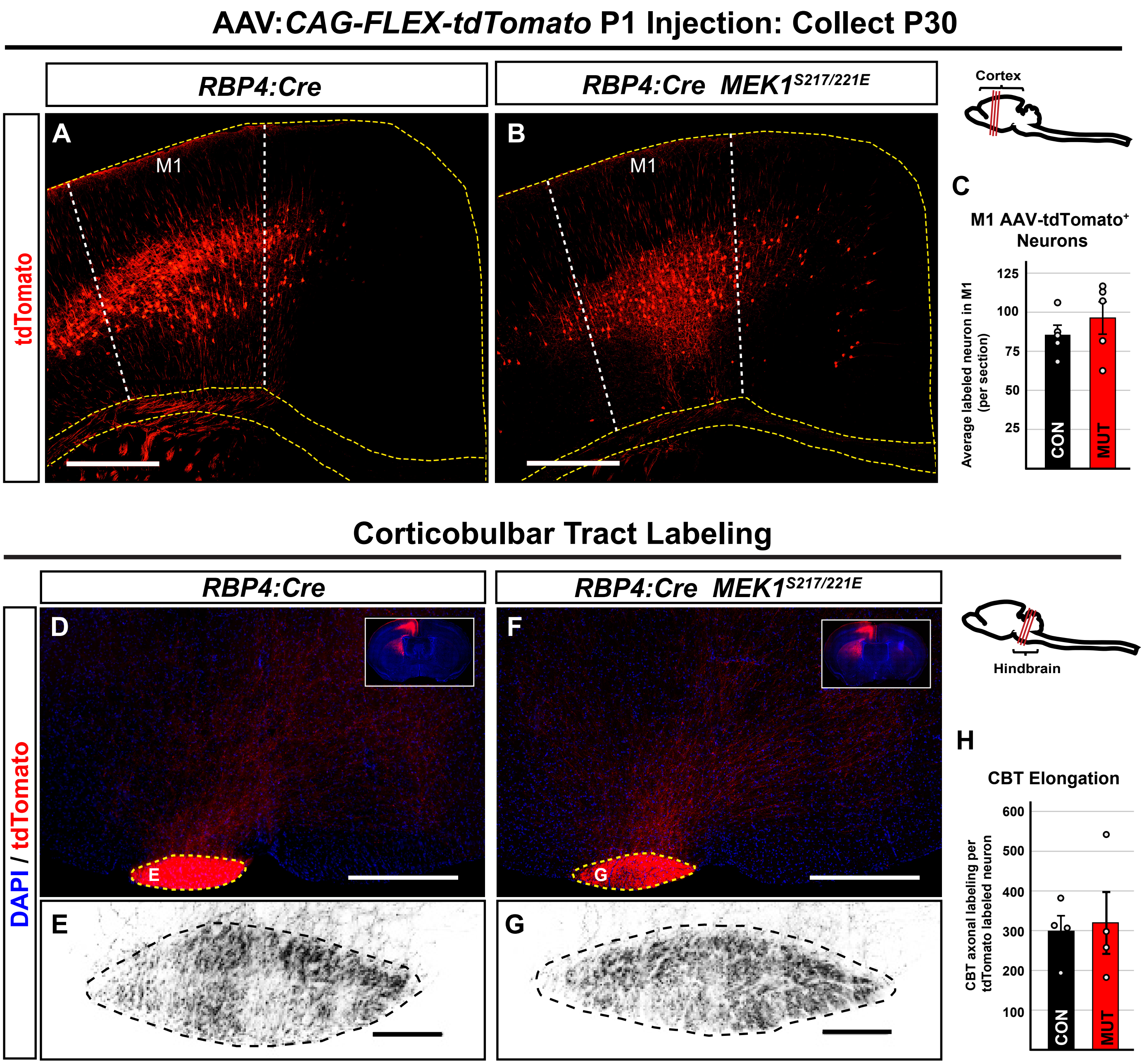

AAV:CAG-FLEX-tdTomato P1 Injection: Collect P30
RBP4:Cre
RBP4:Cre MEK1S217/221E
Cortex
tdTomato
A
B
M1
M1
C
M1 AAV-tdTomato+ Neurons
Average labeled neuron in M1 (per section)
125
100
75
50
25
CON
MUT
Corticobulbar Tract Labeling
RBP4:Cre
RBP4:Cre MEK1S217/221E
Hindbrain
DAPI / tdTomato
D
F
E
G
H
CBT Elongation
CBT axonal labeling per tdTomato labeled neuron
600
500
400
300
200
100
CON
MUT

Fig. S4. Corticobulbar axon extension is not affected in *RBP4:Cre; MEK1*$^{S217/2121E}$ mutants.

A-C. Representative AAV injection sites in M1. AAV-tdTomato labeled cell counts in the motor cortex showed no significant differences between *RBP4:Cre;MEK1*$^{S217/221E}$ (B) and *RBP4:Cre* (A) mice as quantified in C (tdTomato^{+} cells per section, mean ± SEM, n=4, Student's t-test p=0.4). **D-H.** Representative images of corticobulbar tract labeling. Quantification of tdTomato labeled axons in the corticobulbar tract revealed no significant differences between the *RBP4:Cre; MEK1*$^{S217/221E}$ (F-G) and *RBP4:Cre* (D-E) control mice (normalized to M1 labeled neurons, mean ± SEM, n=4, Student's t-test p=0.818) (H).

Scale bars: A,B,D,F = 500µm, E,G= 20µm.

Disease Models & Mechanisms • Supplementary information

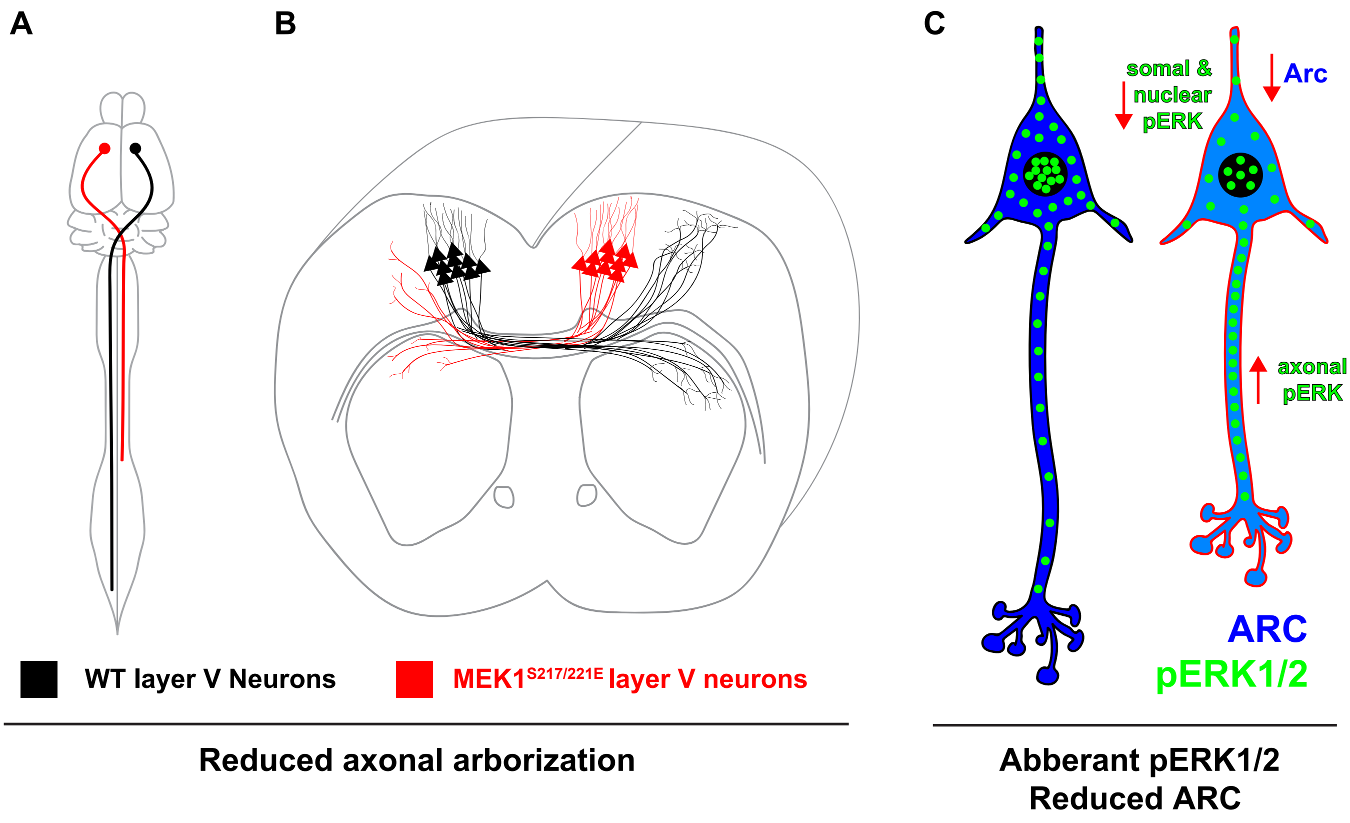

Fig. S5. Model of aberrant circuit development in excitatory neuron-specific *MEK1*$^{217/221E}$ mice.

A-C. *MEK1*$^{S217/221E}$ expressing layer V neurons (red) exhibit excitatory neuron-autonomous reductions in corticospinal axon elongation along the dorsal funiculus (A), reduced arborization in contralateral cortex and striatum (B), and persistent increases in axonal pERK1/2 (C - green dots) when compared to control neurons (black). These axonal deficits in *Nex:Cre; MEK1*$^{S217/221E}$ mutants coincide with decreased levels of pERK1/2 in the soma and nucleus and reduced expression of activity regulated cytoskeleton-associated protein, ARC (blue), which may underly select deficits in motor learning (C).

Disease Models & Mechanisms • Supplementary information
